# Supplementary material for: High phenotypic plasticity at the dawn of the eosauropterygian radiation
Source: PeerJ. 2023 Sep 1;11:e15776. doi: 10.7717/peerj.15776 (PMC10476616; doi:10.7717/peerj.15776)
Supplement: Supplemental Information 1 [file peerj-11-15776-s001.docx]

**High phenotypic plasticity at the dawn of the eosauropterygian radiation**

Antoine Laboury^1^, Torsten M. Scheyer^2^, Nicole Klein^3^, Thomas L. Stubbs^4^, Valentin Fischer^1^

^1^ Evolution & Diversity Dynamics Lab, University of Liège, Liège, Belgium

^2^ Department of Palaeontology, University of Zurich, Zurich, Switzerland

^3^ University of Bonn, Institute of Geosciences, Paleontology, Bonn, Germany

^4^ School of Life, Health & Chemical Sciences, Open University, Milton Keynes, United Kingdom

Corresponding author:

Antoine Laboury^1^

Email address: a.laboury@uliege.be

**Supplementary information**

Institutional abbreviations

**FMNH**, Field Museum of Natural History, Chicago, USA; **GMPKU**, Geological Museum of Peking University, Beijing, China; **GPIT**, Palaeontological Collection of Tübingen University, Tübingen, Germany; **IVPP**, Institute of Vertebrate Paleontology and Paleoanthropy, Chinese Academy of Sciences, Beijing, China; **JLW**, Private collection Jos Lankamp, Borne, The Netherlands; **LPV,** Luoping Vertebrate Collection, Chengdu Institute of Geology and Mineral Resources, Chengdu, China; **MSNM**, Museo di Storia Naturale di Milano, Milan, Italy; **NME**, Natuurmuseum Enschede, Enschede, The Netherlands; **MNHL**, National Museum of Natural History (Naturalis), Leiden, The Netherlands; **NMNS**, National Museum of Natural Science, Taichung, Taiwan, China; **PIMUZ**, Paläontologisches Institut der Universität Zürich, Zürich, Switzerland; **SIPG**, Steinmann-Institut, Paläontologie, Universität Bonn, Germany; **SMNS**, Staatliches Museum für Naturkunde, Stuttgart, Germany; **TWE**, Museum TwentseWelle, Esnchede, The Netherlands; **Wijk**, Material including this abbreviation in the label in located in the NMNHL; **WS**, *Keichousaurus* Museum of Xingyi National Geological Park, Xingyi, Guizhou, China; **ZMNH**, Zheijiang Museum of Natural History, Hangzhou, Zhejiang;

Definition of the ecomorphological traits

1. **Longirostry:** **snout length/ skull length**. **Completeness: 97.1%**
   Characterizes the hydrodynamic potential of the snout such as the amount of drag during the locomotion (Busbey, 1995), the resistance to lateral shaking (Holzman et al., 2012) or the water volume expelled on the closure of the mouth (Mccurry et al., 2017).
2. **Snout shape ratio:** **snout width / skull length. Completeness: 94.3%**Proxy for the size of the potential ingested prey (MacLaren et al., 2022) and for the water volume that need to be expelled during the closure of the mouth (Mccurry et al., 2017).
3. **Jaw robusticity:** **Jaw height at mid-dentigerous length / mandible length. Completeness: 54.3%**

Characterizes the aspect ratio of the jaw and is a proxy of the robustness and the stiffness in the jaw under biting loads (Anderson et al., 2011; Stubbs & Benton, 2016; MacLaren et al., 2017, 2022). The height of the jaw was measured at the mid-dentigerous zone.

1. **Relative symphysial length:** **Symphyseal length / mandible length. Completeness: 57.1%**
   Characterizes the robustness of the anterior portion of the jaw and its resistance during the prey capture (Stubbs & Benton, 2016; Fischer et al., 2017; MacLaren et al., 2017; Bennion et al., 2022).
2. **Functional toothrow length:** **Dentigerous mandible length / mandible length. Completeness: 57.1%**
   Describe the proportion of the jaw which serves to the capture of the potential prey (MacLaren et al., 2017) . Furthermore, differences in the length of the toothrow reflects different strategies in prey capture; a long dental row would indeed increase the variation in bites forces and speed which would result in more functional variability, as reflected by great differences between the anterior and posterior mechanical advantage (Stubbs & Benton, 2016).
3. **Anterior mechanical advantage:** **Distance between the fulcrum and the mid-point of attachment for the adductor muscles on the dorsal surface of the mandible / distance between the fulcrum and the anterior tip of mandible. Completeness: 68.6%**
   The vertebrate mandible can be modelled as a third-order lever (Stubbs & Benton, 2016) . The mechanical advantage (MA) is an estimation of the ability of a system to transfer the muscles force of the mandible to the prey and can be calculated as the ratio of the inlever and the outlever measurements (Stayton, 2006; Stubbs & Benton, 2016). The anterior mechanical advantage (AMA) can be defined as the lowest potential MA along the dental row (Stubbs & Benton, 2016). Here, the inlever is the distance between the fulcrum (skull articulation with the mandible) to the mid-point of the adductor muscles area insertion while the outlever is represented by the distance from the fulcrum to the base of the anteriormost tooth. It has been demonstrated that animals with weak and rapid bites have low values for AMA while higher values are characteristics of powerful and slower bites (Anderson, 2009).
4. **Posterior mechanical advantage:** **Distance between the fulcrum and the mid-point of attachment for the adductor muscles on the dorsal surface of the mandible / distance between the fulcrum and the last mandible tooth. Completeness: 54.3%**

The principle of the posterior mechanical advantage (PMA) is the same than the AMA except that the outlever is measured from the fulcrum to the base of the posteriormost tooth of the mandible. This morphological trait therefore estimates the highest force transfer along the mandibular dentition (Anderson et al., 2011; Stubbs & Benton, 2016).

1. **Opening mechanical advantage:** **Distance between the fulcrum and the retroarticular process / distance between the fulcrum and the mid-point of attachment for the adductor muscles on the dorsal surface of the mandible. Completeness: 82.9%**
   Characterizes the velocity during the opening of the jaw (MacLaren et al., 2017; Bennion et al., 2022). The inlever can be measured as the distance between the fulcrum and the posterior end of retroarticular process and the outlever, as the distance from the fulcrum to the base of the anteriormost tooth of the mandible.
2. **Nares position:** **Distance between the anterior margin of the nares and the tip of the snout / skull length. Completeness: 100%**
   Proxy describing the ability to take a breath during a constant state swim (MacLaren et al., 2022).
3. **Relative naris size: Length of the nares / skull length. Completeness: 100%**Amount of air that can be inhaled during a breath (MacLaren et al., 2022).
4. **Relative orbit size: Mean diameter of the orbit / skull length. Completeness: 97.1%**
   Characterizes the importance of the visual component in the multiple functions that skulls have to perform (MacLaren et al., 2022).
5. **Occular offset:** **Distance from the centre of the orbit to the plane containing the upper tooth row / skull length. Completeness: 51.4%**
   Characterizes the position of the eyes in relation to the jaws.
6. **Relative parietal foramen length:** **Length of the parietal foramen / skull length. Completeness: 100%**
   Characterizes the length of the parietal foramen and is a proxy for the relative size of the pineal gland (MacLaren et al., 2022).
7. **Tooth crown shape:** **Tooth crown height / crown base width. Completeness: 97.1%**
   Characterizes the slenderness of the tooth and the resistance to bite forces (Massare, 1987; Foffa et al., 2018; Zverkov et al., 2018; MacLaren et al., 2022; Fischer et al., 2022). Measurements have been taken on mid-dentigerous region and not on fangs.
8. **Absolute crown height:** **Tooth crown height raw measurement. Completeness: 100%**
   This absolute trait has been shown to be a determinant of diet in modern cetaceans (Ridgway & Harrison, 1999) and have been recently used in marine reptile dataset (Fischer et al., 2020; Bennion et al., 2022).
9. **Heterodonty index:** **Anterior tooth crown shape / posterior tooth crown shape. Completeness: 82.9%**
   Characterizes quantitatively the level of heterodonty in the dentition of taxa. A value close to 1 indicates no or very low level of heterodonty while a value which exceed 1 reflects a certain level of heterodonty. This quantitative trait however doesn’t inform about the global morphology of the teeth should therefore be used in combinaison with discrete character as 18, 19, 20 which describe the morphology of the tooth crown.
10. **Jaw or snout anterior constriction: Absent (0); Present (1). Completeness: 94.3%**
    Discrete character which represents a proxy for the preferential use of the anterior jaw for prey capture (MacLaren et al., 2022).
11. **Pointed and recurved tooth crown: Absent (0); Present (1). Completeness: 94.3%**
    Discrete character which serves to differentiate the small-prey flesh piercers or cutters which have pointed recurved crowns than from the taxa which has straight or bulbous crowns with blunt or rounded apices and which could be referred in the durophage or generalist guilds in Massare, (1987) and Fischer et al. (2022).
12. **Bulbous crushing dentition: Absent (0); Present (1). Completeness: 100%**
    Discrete character which serves to differentiate taxa with bulbous, blunt and rounded (globidont) dentition. This dental feature is characteristic of the durophage guild made up with taxa capable of crushing hard-shelled prey (Massare, 1987; Fischer et al., 2022). Please note that *Placodus* and cyamodontoid placodonts which are highly durophageous taxa have a rather flat instead of bulbous dentition (Neenan et al., 2013).
13. **Enlarge procumbent dentition: Absent (0); Present (1)**. **Completeness: 100%**
    Discrete character which serves to differentiate taxa that possess fangs on lower and upper jaws. These fangs would have served to pierce pelagic preys (Massare, 1987; Grossman, 2007) or to create a fish-trap to prevent preys from escaping out of the buccal cavity (Rieppel, 2002).
14. **Relative skull length:** **Skull length / trunk length. Completeness: 60%**
    Characterizes the proportion of the body used for prey capture and sensory capabilities.
15. **Neck proportion:** **Neck length / trunk length. Completeness: 60%**
    Characterizes the general body plan but is also a proxy for potential feeding arch and reach of cranium away from the trunk (O’Keefe, 2001).
16. **Trunk proportion:** **Trunk length / body length. Completeness: 40%**
    Characterizes the proportion of the body length available for lungs and digestive track.
17. **Tail proportion:** **Tail length / body length. Completeness: 40%**
    Characterizes the proportion of the body used for the locomotion. Because Plesiosauria evolved to an undewater flight by using their four hydrofoil-like flippers (Krahl, 2021), the role of the tail in the locomotion is greatly reduced as evidenced by the shortening of the tail (Krahl, 2021). However, the stem groups Pachypleurosauroidea and Nothosauroidea are regarded as lateral undulatory swimmers even if the fore- and the hindflippers played an important role in the propulsion as there are morphologically derived (Carroll, Gaskill & Whittington, 1985; Sues, 1987; Krahl, 2021).
18. **Propodial variation:** **Humerus proximo-distal length / femur proximodistal length. Completeness: Completeness: 77.1%**Characterizes the appendicular body plan. The length of the humerus and the femur relative to each other can be considered as indicative of forelimb, hindlimb or equally driven underwater locomotion (Zeffer, Johansson & Marmebro, 2003; Hinić-Frlog & Motani, 2010).
19. **Propodial size:** **Humerus proximo-distal length / skull length. Completeness: 77.1%**
    Characterizes the elongation of the limbs relative to the skull size. This trait is indicative of the ecology of taxa as species with long limbs and small head will swim and feed in a different way than taxa with a small limbs and large head (Taylor, 1987; Massare, 1988).
20. **Humerus gracility:** **Humerus antero-posterior width / humerus proximo-distal length. Completeness: 85.7%**
    Characterizes the area of muscular insertion on the humerus and the mechanical efficiency of the pectoral girdle. This trait describes the implication of the forelimb in the locomotion.
21. **Femur gracility:** **Femur antero-posterior width / femur proximo-distal length. Completeness: 80%**
    Characterizes the area of muscular insertion on the femur and the mechanical efficiency of the pelvic girdle. This trait describes the implication of the forelimb in the locomotion.
22. **Forelimb aspect ratio**: **Forelimb antero-posterior width / forelimb proximo-distal length. Completeness: 51.4%**
    Describes potential water displacement by the forelimbs or the pectoral paddles.
23. **Hindlimb aspect ratio:** **Hindlimb antero-posterior width / forelimb proximo-distal length. Completeness: 62.9%**
    Describes potential water displacement by the hindlimbs or the pelvic paddles.

Supplementary tables

**Table S1** List of specimens present in the analyses and data sources.

| Taxon | Clade | Locality | Age range | Source of measurements |
| --- | --- | --- | --- | --- |
| *Anarosaurus heterodontus* | Pachypleurosauroidea | Western Tethys | 246.36­ – 243.99 | First-hand examination and photographs (Klein, 2009; 2012) |
| Dawazisaurus brevis | Pachypleurosauroidea | Eastern Tethys | 244.94 – 243.99 | Photographs (Cheng *et al.,* 2016) |
| *Diandongosaurus acutidentatus* | Pachypleurosauroidea | Eastern Tethys | 244.94 – 243.99 | Photographs (Shang *et al*., 2011 and Sato *et al.,* 2013) |
| *Dianmeiosaurus gracilis* | Pachypleurosauroidea | Eastern Tethys | 244.94 – 243.99 | Photographs (Shang & Li, 2015) |
| *Dianopachysaurus dingi* | Pachypleurosauroidea | Eastern Tethys | 244.94 – 243.99 | Photographs (Liu *et al.,* 2011) |
| *Honghesaurus longicaudalis* | Pachypleurosauroidea | Eastern Tethys | 244.94 – 243.99 | Photographs (Xu *et al.,* 2022) |
| *Luopingosaurus*  *imparilis* | Pachypleurosauroidea | Eastern Tethys | 244.94 – 243.99 | Photographs (Xu *et al.,* 2023) |
| *Keichousaurus hui* | Pachypleurosauroidea | Eastern Tethys | 239.1 – 237 | First-hand examination and photographs (Holmes & Cheng, 2008) |
| *Neusticosaurus edwardsii* | Pachypleurosauroidea | Western Tethys | 239.1 – 237 | First-hand examination |
| *Neusticosaurus peyeri* | Pachypleurosauroidea | Western Tethys | 241.5 – 239.1 | First-hand examination |
| *Neusticosaurus pusillus* | Pachypleurosauroidea | Western Tethys | 241.5 – 239.1 | First-hand examination |
| *Odoiporosaurus teruzzi* | Pachypleurosauroidea | Western Tethys | 243.99 – 241.5 | Photographs (Renesto *et al.,* 2014) |
| *Panzhousaurus rotundirostris* | Pachypleurosauroidea | Eastern Tethys | 244.94 – 243.99 | Photographs (Jiang *et al.,* 2019) |
| *Prosantosaurus scheffoldi* | Pachypleurosauroidea | Western Tethys | 241.5 – 239.1 | First-hand examination |
| *Qianxisaurus chajiangensis* | Pachypleurosauroidea | Eastern Tethys | 239.1 – 237 | Photographs (Cheng *et al.,* 2012) |
| *Serpianosaurus mirigiolensis* | Pachypleurosauroidea | Western Tethys | 243.99 – 241.5 | First-hand examination |
| *Wumengosaurus delicatomandibularis* | Pachypleurosauroidea | Eastern Tethys | 244.94 – 243.99 | Photographs (Jiang *et al.,* 2008 and Wu *et al.,* 2020) |
| *Brevicaudosaurus jiyangshanensis* | Nothosauroidea | Eastern Tethys | 239.1 – 237 | Photographs (Shang *et al.,* 2020) |
| *Lariosaurus calcagnii* | Nothosauroidea | Western Tethys | 241.5 – 239.1 | First-hand examination |
| *Lariosaurus balsami* | Nothosauroidea | Western Tethys | 239.1 – 237 | First-hand examination |
| *Lariosaurus buzzii* | Nothosauroidea | Western Tethys | 243.99 – 241.5 | First-hand examination |
| *Lariosaurus hongguoensis* | Nothosauroidea | Eastern Tethys | 244.94 – 243.99 | Photographs (Jiang *et al.,* 2006) |
| *Lariosaurus xingyensis* | Nothosauroidea | Eastern Tethys | 239.1 – 237 | Photographs (Rieppel *et al.,* 2003 and Lin *et al.* 2017) |
| *Lariosaurus vosseveldensis* | Nothosauroidea | Western Tethys | 246.36­ – 243.99 | Photographs (Klein *et al.,* 2016) |
| *Lariosaurus winkelhorsti* | Nothosauroidea | Western Tethys | 246.36 – 244.94 | First-hand examination, photographs made by the authors |
| *Lariosaurus youngi* | Nothosauroidea | Eastern Tethys | 239.1 – 237 | Photographs (Ji *et al.,* 2014) |
| *Nothosaurus cristatus* | Nothosauroidea | Western Tethys | 239.1 – 237 | First-hand examination |
| *Nothosaurus luopingensis* | Nothosauroidea | Eastern Tethys | 244.94 – 243.99 | Photographs (Shang *et al.,* 2022) |
| *Nothosaurus giganteus* | Nothosauroidea | Western Tethys | 243.99 – 233.5 | First-hand examination and 3D models created by the authors |
| *Nothosaurus jagisteus* | Nothosauroidea | Western Tethys | 241.5 – 239.1 | First-hand examination |
| *Nothosaurus marchicus* | Nothosauroidea | Western Tethys | 246.5 – 241.5 | First-hand examination, photographs (Klein *et al.,* 2015 and Voeten *et al.,* 2018) |
| *Nothosaurus mirabilis* | Nothosauroidea | Western Tethys | 243.99 – 239.1 | First-hand examination and 3D models created by the authors |
| *Simosaurus gaillardoti* | Nothosauroidea | Western Tethys | 241.5 – 237 | 3D models created by the authors |
| *Augustasaurus hagdorni* | Pistosauroidea | Eastern Panthalassa | 243.99 – 241.5 | 3D models created by the authors |
| *Wangosaurus brevirostris* | Pistosauroidea | Eastern Tethys | 239.1 – 237 | Photographs (Ma *et al.,* 2015) |
| *Yunguisaurus liae* | Pistosauroidea | Eastern Tethys | 239.1 – 237 | Photographs (Cheng *et al.,* 2006 and Sato *et al.,* 2014) |

**Table S2:** List of specimens and completeness for all morphological regions.

| Taxon | Clade | Specimens | Craniodental completeness | Postcranial  completeness | Whole body completeness |
| --- | --- | --- | --- | --- | --- |
| *Anarosaurus heterodontus* | Pachypleurosauroidea | NME 480000125, NME 480000130, NMNHL RGM 443855, NMNHL RGM 443856, SIPG R 594, SIPG R 595, SIPG R 596, NMNHL Wijk06-38, NMNHL Wij06-266, NMNHL Wijk09-582, NMNHL RGM.443858 | 95.24% | 30% | 75% |
| *Dawazisaurus brevis* | Pachypleurosauroidea | NMNS000933-F034397 | 90.48% | 100% | 93.75% |
| *Diandongosaurus acutidentatus* | Pachypleurosauroidea | IVPP V17760, NMNS-000933-F03498 | 95.24% | 100% | 96.88% |
| *Dianmeiosaurus gracilis* | Pachypleurosauroidea | IVPP V 17054, IVPP V 18630 | 90.48% | 100% | 93.75% |
| *Dianopachysaurus dingi* | Pachypleurosauroidea | LPV 31365 | 66.67% | 60% | 65.63% |
| *Honghesaurus longicaudalis* | Pachypleurosauroidea | IVPP V30380 | 95.24% | 100% | 96.88% |
| *Luopingosaurus*  *imparilis* | Pachypleurosauroidea | IVPP V19049 | 80.95% | 80% | 81.25% |
| *Keichousaurus hui* | Pachypleurosauroidea | NMNS-cyn-2003-25, NMNS-cyn-2005-05, NMNS-cyn-2005-12, SMNS 81780, SMNS 59705 | 95.24% | 100% | 96.88% |
| *Neusticosaurus edwardsii* | Pachypleurosauroidea | PIMUZ T2810, PIMUZ T2811, PIMUZ T3430, PIMUZ T3439, PIMUZ T3453, PIMUZ T3452, PIMUZ T3460, PIMUZ T3708, PIMUZ T3758, PIMUZ T3759, PIMUZ T3776, PIMUZ T4761 | 100% | 100% | 100 % |
| *Neusticosaurus peyeri* | Pachypleurosauroidea | PIMUZ T3393, PIMUZ T3394, PIMUZ T3395, PIMUZ T3396, PIMUZ T3403, PIMUZ T3410, PIMUZ T3422, PIMUZ T3423, PIMUZ T3431, PIMUZ T3445, PIMUZ T3461, PIMUZ T3464,  PIMUZ T3467, PIMUZ T3474, PIMUZ T3476, PIMUZ T3479, PIMUZ T3497, PIMUZ T3511, PIMUZ T3542, PIMUZ T3546, PIMUZ T3607, PIMUZ T3710, PIMUZ T3728, PIMUZ T3744, PIMUZ T3902 | 100% | 100% | 100% |
| *Neusticosaurus pusillus* | Pachypleurosauroidea | PIMUZ T3902, PIMUZ T3400,  PIMUZ T3421, PIMUZ T3426, PIMUZ T3429, PIMUZ T3442, PIMUZ T3468, PIMUZ T3509, PIMUZ T3530, PIMUZ T3536, PIMUZ T3538, PIMUZ T3547, PIMUZ T3556, PIMUZ T3574, PIMUZ T3598, PIMUZ T3601, PIMUZ T3604, PIMUZ T3605, PIMUZ T3612, PIMUZ T3614, PIMUZ T3625, PIMUZ T3627, PIMUZ T3639, PIMUZ T3649 A, PIMUZ T3649 B, PIMUZ T3649 C, PIMUZ T3653, PIMUZ T3654, PIMUZ T3658, PIMUZ T3658, PIMUZ T3671, PIMUZ T 3672, PIMUZ T3703, PIMUZ T3739, PIMUZ T3741 B, PIMUZ T3803 B, PIMUZ T3803 D, PIMUZ T3934, PIMUZ T4289, PIMUZ T5942 | 100% | 100% | 100% |
| *Odoiporosaurus teruzzi* | Pachypleurosauroidea | MSNM BES SC 1893 | 85.71% | 40% | 71.88% |
| *Panzhousaurus rotundirostris* | Pachypleurosauroidea | GMPKU-P- 1059 | 66.67% | 80% | 71.88% |
| *Prosantosaurus scheffoldi* | Pachypleurosauroidea | PIMUZ A/III 1197, PIMUZ A/III 1240, PIMUZ A/III 1273, PIMUZ A/III 1274, PIMUZ A/III 1275, PIMUZ A/III 4566 | 95.24% | 80% | 90.63% |
| *Qianxisaurus chajiangensis* | Pachypleurosauroidea | NMNS-KIKO-F044630 | 90.48% | 60% | 81.25% |
| *Serpianosaurus mirigiolensis* | Pachypleurosauroidea | PIMUZ T96, PIMUZ T951, PIMUZ T1071, PIMUZ T 3675, PIMUZ T3676, PIMUZ T3677, PIMUZ T3680, PIMUZ T3685, PIMUZ T3742, PIMUZ T3931, PIMUZ T3933 | 100% | 100% | 100% |
| *Wumengosaurus delicatomandibularis* | Pachypleurosauroidea | GMPKU-P-1210, IVPP V15314, NMNS-KIKO-F071129-Z, ZMNH M8758 | 90.48% | 100% | 93.75% |
| *Brevicaudosaurus jiyangshanensis* | Nothosauroidea | IVPP V 18625 | 76.19% | 100% | 84.38% |
| *Lariosaurus balsami* | Nothosauroidea | PIMUZ T4856 | 80.95% | 80% | 81.25% |
| *Lariosaurus buzzii* | Nothosauroidea | PIMUZ T2804 | 76.19% | 40% | 65.63% |
| *Lariosaurus calcagnii* | Nothosauroidea | PIMUZ T2460, PIMUZ T2461, PIMUZ T2462, PIMUZ T2464, PIMUZ T4836, PIMUZ T5151, PIMUZ T5559 | 95.24% | 100% | 96.88% |
| *Lariosaurus hongguoensis* | Nothosauroidea | GMPKU-P-1011 | 85.71% | 50% | 75% |
| *Lariosaurus vosseveldensis* | Nothosauroidea | TWE 480000504 | 66.67% | 0% | 46.88% |
| *Lariosaurus youngi* | Nothosauroidea | WS-30-R24 | 80.95% | 80% | 81.25% |
| *Nothosaurus cristatus* | Nothosauroidea | GPIT-PV-75067 | 66.67% | 0% | 46.88% |
| *Nothosaurus luopingensis* | Nothosauroidea | IVPP V 24895 | 66.67% | 60% | 65.63% |
| *Nothosaurus giganteus* | Nothosauroidea | PIMUZ T4829; SMNS 18058, SMNS 57047, SMNS 80217, SMNS 1598b ; SMNS 159157, SMNS 17822c, SMNS 81311 | 95.24% | 80% | 90.63% |
| *Nothosaurus jagisteus* | Nothosauroidea | SMNS 56618 | 66.67% | 10% | 50% |
| *Nothosaurus marchicus* | Nothosauroidea | JLW 300, NMNHL RGM 449995, TWE 480000375, TWE 4800000473, TWE 4800000474 | 85.71% | 30% | 68.75% |
| *Nothosaurus mirabilis* | Nothosauroidea | SMNS 13155, SMNS 15714, SMNS 16433, SMNS 56826, SMNS 59074, SMNS 84550 | 71.43% | 10% | 53.13% |
| *Nothosaurus winkelhorsti* | Nothosauroidea | TWE 4800000474 | 57.14% | 0% | 40.63% |
| *Simosaurus gaillardoti* | Nothosauroidea | GPIT-PV-60638, SMNS 10360, SMNS 16363, SMNS16638, SMNS 50714, SMNS 59366, SMNS 7861, SMNS 17223, SMNS 14733, SMNS 17590 | 100% | 10% | 71.88% |
| *Augustasaurus hagdorni* | Pistosauroidea | FMNH PR1974 | 85.71% | 0% | 59.38% |
| *Wangosaurus brevirostris* | Pistosauroidea | GMPKU-P-1529 | 80.95% | 70% | 78.13% |
| *Yunguisaurus liae* | Pistosauroidea | IVPP V14993, NMNS 004529/F003826, ZMNH M8738 | 95.24% | 100% | 96.88% |

**Table S3:** Results of the principal coordinate analysis (PCoA) on whole body data.

|  | Eigenvalues | Corrected eigenvalues | Relative corrected eigenvalues | Cumulative relative eigenvalues |
| --- | --- | --- | --- | --- |
| 1 | 0.900819113 | 2.88434764 | 0.227667249 | 0.2276672 |
| 2 | 0.253070429 | 0.95844403 | 0.075651878 | 0.3033191 |
| 3 | 0.162792816 | 0.77097610 | 0.060854665 | 0.3641738 |
| 4 | 0.147754911 | 0.66208497 | 0.052259673 | 0.4164335 |
| 5 | 0.091958429 | 0.51555236 | 0.040693565 | 0.4571270 |
| 6 | 0.075020194 | 0.45230782 | 0.035701549 | 0.4928286 |
| 7 | 0.066492483 | 0.42775950 | 0.033763901 | 0.5265925 |
| 8 | 0.055621819 | 0.39871364 | 0.031471254 | 0.5580637 |
| 9 | 0.049897814 | 0.36862100 | 0.029095982 | 0.5871597 |
| 10 | 0.041485684 | 0.36173152 | 0.028552182 | 0.6157119 |
| 11 | 0.029440052 | 0.32360907 | 0.025543102 | 0.6412550 |
| 12 | 0.026604858 | 0.31021024 | 0.024485506 | 0.6657405 |
| 13 | 0.020967539 | 0.30258431 | 0.023883576 | 0.6896241 |
| 14 | 0.014470059 | 0.27043585 | 0.021346036 | 0.7109701 |
| 15 | 0.012796317 | 0.25735946 | 0.020313890 | 0.7312840 |
| 16 | 0.007406053 | 0.24513570 | 0.019349044 | 0.7506331 |
| 17 | 0.005784011 | 0.24116281 | 0.019035456 | 0.7696685 |
| 18 | 0.003548252 | 0.22981656 | 0.018139874 | 0.7878084 |
| 19 | 0.001751484 | 0.21709621 | 0.017135832 | 0.8049442 |
| 20 | 0.001219537 | 0.20746838 | 0.016375889 | 0.8213201 |
| 21 | 0.000000000 | 0.20417877 | 0.016116233 | 0.8374363 |
| 22 | -0.001999835 | 0.20169884 | 0.015920488 | 0.8533568 |
| 23 | -0.006028007 | 0.19743376 | 0.015583836 | 0.8689407 |
| 24 | -0.006877943 | 0.19077044 | 0.015057887 | 0.8839985 |
| 25 | -0.007477833 | 0.18682552 | 0.014746507 | 0.8987451 |
| 26 | -0.009552126 | 0.17893014 | 0.014123309 | 0.9128684 |
| 27 | -0.013605055 | 0.17420866 | 0.013750633 | 0.9266190 |
| 28 | -0.014301976 | 0.16203741 | 0.012789932 | 0.9394089 |
| 29 | -0.019871359 | 0.15605509 | 0.012317736 | 0.9517267 |
| 30 | -0.021004283 | 0.15057245 | 0.011884981 | 0.9636116 |
| 31 | -0.025431855 | 0.14364793 | 0.011338415 | 0.9749501 |
| 32 | -0.029774570 | 0.12297443 | 0.009706615 | 0.9846567 |
| 33 | -0.034917974 | 0.11531397 | 0.009101959 | 0.9937586 |
| 34 | -0.042399820 | 0.07907271 | 0.006241365 | 1.0000000 |
| 35 | -0.060260617 | 0.00000000 | 0.000000000 | 1.0000000 |
| 36 | -0.087378534 | 0.00000000 | 0.000000000 | 1.0000000 |

**Table S3:** Results of the principal coordinate analysis (PCoA) on craniodental data.

|  | Eigenvalues | Corrected eigenvalues | Relative corrected eigenvalues | Cumulative relative eigenvalues |
| --- | --- | --- | --- | --- |
| 1 | 1.2635676570 | 3.50154136 | 0.301730694 | 0.3017307 |
| 2 | 0.3034982495 | 0.98848790 | 0.085178814 | 0.3869095 |
| 3 | 0.2118989408 | 0.78149544 | 0.067342104 | 0.4542516 |
| 4 | 0.1346167277 | 0.55801617 | 0.048084712 | 0.5023363 |
| 5 | 0.0994765175 | 0.45914471 | 0.039564877 | 0.5419012 |
| 6 | 0.0840919306 | 0.39065275 | 0.033662868 | 0.5755641 |
| 7 | 0.0687569192 | 0.37609500 | 0.032408415 | 0.6079725 |
| 8 | 0.0474408684 | 0.31918422 | 0.027504366 | 0.6354768 |
| 9 | 0.0375529728 | 0.28246369 | 0.024340128 | 0.6598170 |
| 10 | 0.0326520361 | 0.26763897 | 0.023062670 | 0.6828796 |
| 11 | 0.0264576634 | 0.25177152 | 0.021695359 | 0.7045750 |
| 12 | 0.0180770722 | 0.23364120 | 0.020133054 | 0.7247081 |
| 13 | 0.0133468761 | 0.22302871 | 0.019218567 | 0.7439266 |
| 14 | 0.0119916478 | 0.20460528 | 0.017631005 | 0.7615576 |
| 15 | 0.0070750902 | 0.19867522 | 0.017120007 | 0.7786776 |
| 16 | 0.0036369317 | 0.19058981 | 0.016423280 | 0.7951009 |
| 17 | 0.0020655508 | 0.18123259 | 0.015616961 | 0.8107179 |
| 18 | 0.0007128037 | 0.17046664 | 0.014689250 | 0.8254071 |
| 19 | 0.0000000000 | 0.15906745 | 0.013706973 | 0.8391141 |
| 20 | -0.0005043823 | 0.15799045 | 0.013614167 | 0.8527283 |
| 21 | -0.0015741677 | 0.15102427 | 0.013013886 | 0.8657422 |
| 22 | -0.0023127154 | 0.14972141 | 0.012901617 | 0.8786438 |
| 23 | -0.0041367266 | 0.14744869 | 0.012705775 | 0.8913495 |
| 24 | -0.0060633665 | 0.14235979 | 0.012267260 | 0.9036168 |
| 25 | -0.0085828018 | 0.13945009 | 0.012016529 | 0.9156333 |
| 26 | -0.0115292104 | 0.13400741 | 0.011547529 | 0.9271809 |
| 27 | -0.0129376477 | 0.12973957 | 0.011179765 | 0.9383606 |
| 28 | -0.0153761066 | 0.12550170 | 0.010814585 | 0.9491752 |
| 29 | -0.0202281418 | 0.12164442 | 0.010482200 | 0.9596574 |
| 30 | -0.0225841714 | 0.11592881 | 0.009989680 | 0.9696471 |
| 31 | -0.0238780446 | 0.10613917 | 0.009146099 | 0.9787932 |
| 32 | -0.0297713334 | 0.10360215 | 0.008927482 | 0.9877207 |
| 33 | -0.0350823809 | 0.07670943 | 0.006610114 | 0.9943308 |
| 34 | -0.0403180352 | 0.06579036 | 0.005669210 | 1.0000000 |
| 35 | -0.0562765818 | 0.00000000 | 0.00000000 | 1.0000000 |
| 36 | -0.0898056789 | 0.00000000 | 0.00000000 | 1.0000000 |

**Table S3:** Results of the principal coordinate analysis (PCoA) on postcranial data.

|  | Eigenvalues | Corrected eigenvalues | Relative corrected eigenvalues | Cumulative relative eigenvalues |
| --- | --- | --- | --- | --- |
| 1 | 110.83622563 | 261.989699 | 0.201167857 | 0.2011679 |
| 2 | 61.39924371 | 169.250088 | 0.129958077 | 0.3311259 |
| 3 | 37.98120429 | 113.994146 | 0.087529999 | 0.4186559 |
| 4 | 28.95846170 | 92.634781 | 0.071129287 | 0.4897852 |
| 5 | 20.80855263 | 77.743216 | 0.059694851 | 0.5494801 |
| 6 | 16.08398249 | 66.131734 | 0.050779016 | 0.6002591 |
| 7 | 9.25046506 | 53.013631 | 0.040706328 | 0.6409654 |
| 8 | 7.56236299 | 46.888193 | 0.036002932 | 0.6769683 |
| 9 | 6.58872541 | 43.230154 | 0.033194120 | 0.7101625 |
| 10 | 3.11778978 | 37.154685 | 0.028529093 | 0.7386916 |
| 11 | 2.76509109 | 33.869918 | 0.026006896 | 0.7646985 |
| 12 | 1.81325068 | 33.584659 | 0.025787861 | 0.7904863 |
| 13 | 0.52345718 | 31.010514 | 0.023811313 | 0.8142976 |
| 14 | 0.26629687 | 28.505894 | 0.021888149 | 0.8361858 |
| 15 | 0.00000000 | 26.893402 | 0.020650003 | 0.8568358 |
| 16 | -0.02374094 | 25.812694 | 0.019820185 | 0.8766560 |
| 17 | -0.16959535 | 24.085404 | 0.018493892 | 0.8951499 |
| 18 | -0.22324129 | 23.858587 | 0.018319731 | 0.9134696 |
| 19 | -0.85615380 | 23.536235 | 0.018072215 | 0.9315418 |
| 20 | -1.02261565 | 20.985369 | 0.016113541 | 0.9476553 |
| 21 | -1.53274510 | 19.858658 | 0.015248400 | 0.9629037 |
| 22 | -1.71927242 | 17.644080 | 0.013547944 | 0.9764517 |
| 23 | -3.23549694 | 15.908408 | 0.012215214 | 0.9886669 |
| 24 | -4.25828592 | 9.510221 | 0.007302389 | 0.9959693 |
| 25 | -5.70850431 | 5.249367 | 0.004030708 | 1.0000000 |
| 26 | -9.20200805 | 0.000000 | 0.000000 | 1.0000000 |
| 27 | -14.68643412 | 0.000000 | 0.000000 | 1.0000000 |

****Supplementary figures

**Supplementary figure 1:** Most parsimonious tree arising from the implied weighting analysis (*k*=12). The Consistency Index is 0.310 and the Retention Index is 0.664. The dataset is the same than the original matrix of Xu et al*.* (2022) and no further taxa have been added. As we used the implied weighting algorithm, our topology shows slightly differences with the most parsimonious consensus tree of Xu et al*.* (2022). For example, in our analyse, *Honghesaurus* is no longer the sister taxon of *Wumengosaurus* as *Majiashanosaurus* occupies a more derived position. Values of the symmetric resampling ≥ 50 are indicated at their corresponding nodes.

**Supplementary figure 2:** Time-scaled phylogeny arising from implied weighting (*k*=12) maximum parsimony analysis using minimum branch length algorithm, using a minimal value of 0.5 Myr. Only taxa present in our morphological dataset are shown. Ranges extensions of taxa have been coloured according to their respective clades.

**Supplementary figure 3:** Eosauropterygian quantitative craniodental trait histograms, pairwise distribution and correlation. Each dot is coloured with respect to their relative clade. The upper panel indicates the pairwise correlation (Pearson’s correlation coefficient; * indicates significance at alpha = 0.05, ** at alpha 0.01).

**Supplementary figure 4:** Eosauropterygian quantitative postcranial trait histograms, pairwise distribution and correlation. Each dot is coloured with respect to their relative clade. The upper panel indicates the pairwise correlation (Pearson’s correlation coefficient; * indicates significance at alpha = 0.05, ** at alpha 0.01).

**Supplementary figure 5:** Tanglegram comparing the phylogeny with the hierarchy of a cluster dendrogram generated with the whole-body data. Each label has been coloured with respect to its relative clade. The phylogenetic tree has been generated in Maximum Parsimony framework under implied weighting (*k*=12).

**Supplementary figure 6:** NMDS whole body stress plot, showing the relationship between the dissimilarities between taxa present in the dissimilarity matrix and the ordination on the NMDS morphospace (maximum number of random starts = 100).

**
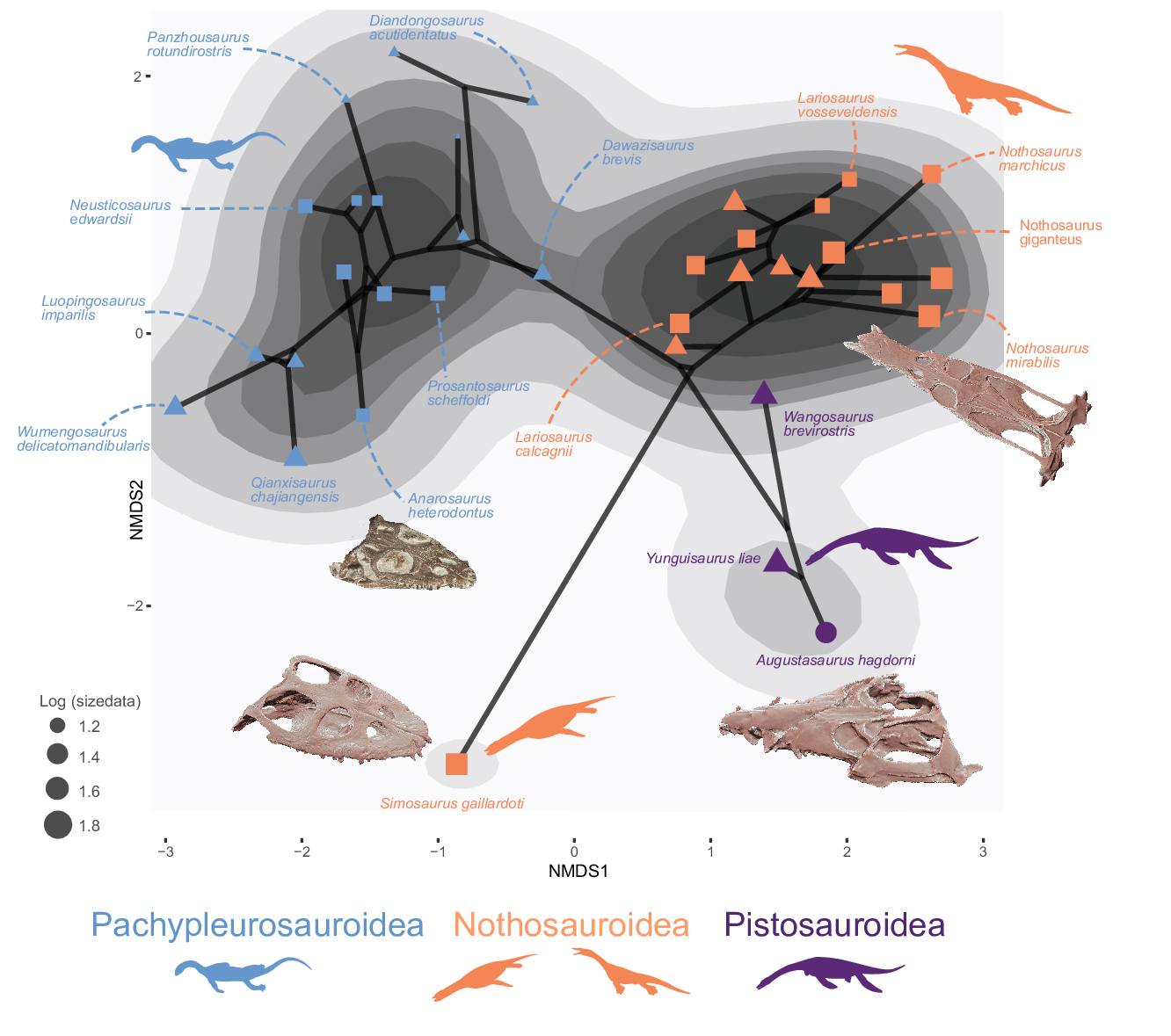
**

**Supplementary figure 7:** Morphospace using the NMDS (dimension=2) on whole body dataset.

**Supplementary figure 8:** NMDS craniodental stress plot, showing the relationship between the dissimilarities between taxa present in the craniodental dissimilarity matrix and the ordination on the NMDS morphospace (maximum number of random starts = 100).


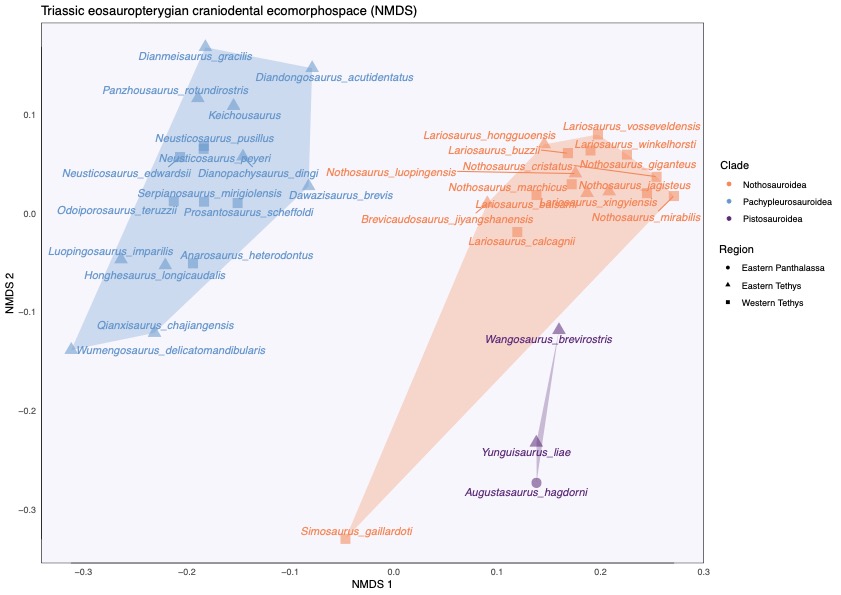


**Supplementary figure 9:** Morphospace using the NMDS (dimension=2) on the craniodental dataset.

**
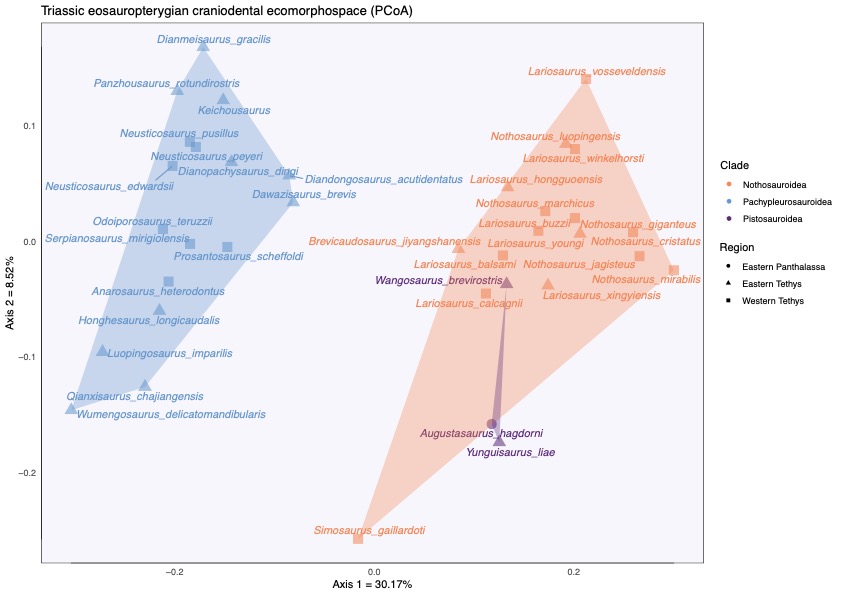
**

**Supplementary figure 10:** Morphospace using the first two axes of the PCoA on the craniodental dataset.

**Supplementary figure 11:** NMDS postcranial stress plot, showing the relationship between the dissimilarities between taxa present in the craniodental dissimilarity matrix and the ordination on the NMDS morphospace (maximum number of random starts = 100).


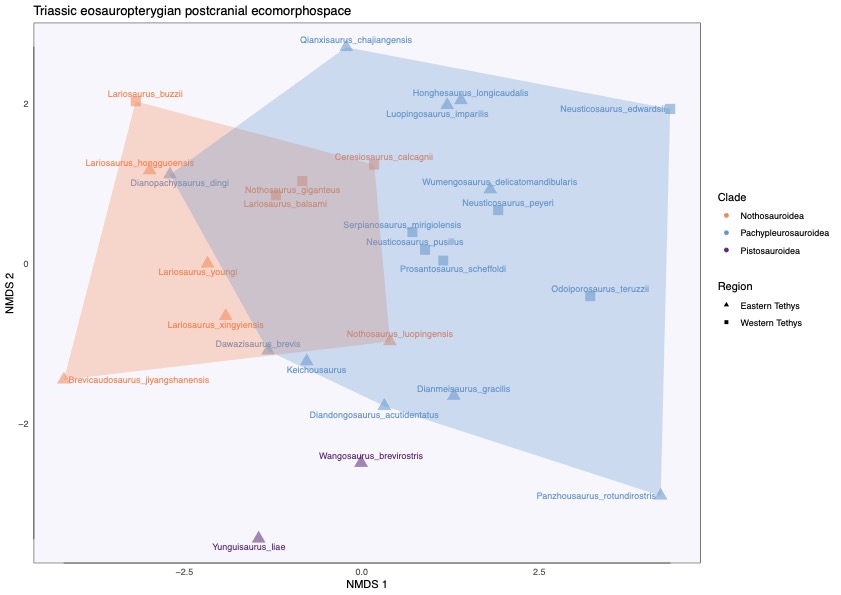


**Supplementary figure 12:** Morphospace using the NMDS (dimension=2) on the postcranial dataset.

**
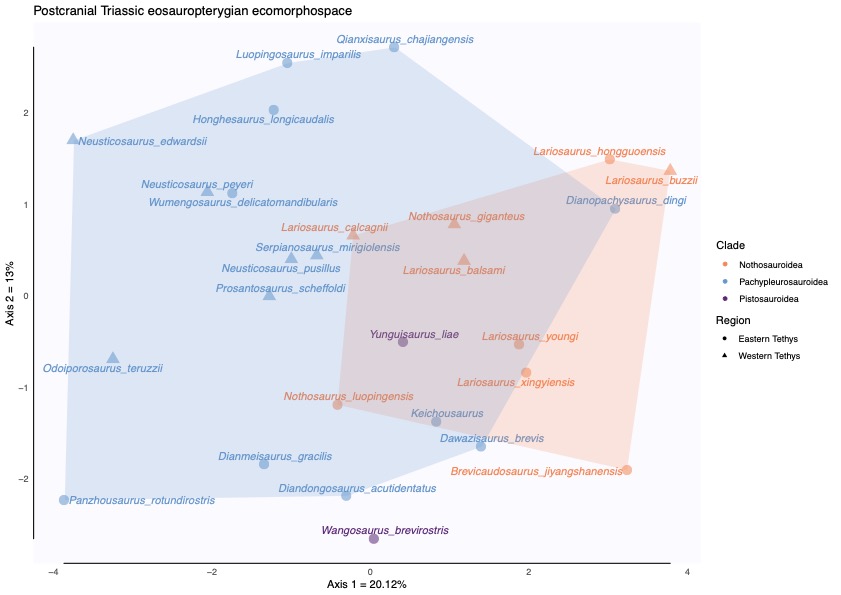
**

**Supplementary figure 13:** Morphospace using the first two axes of the PCoA on the postcranial dataset.

**
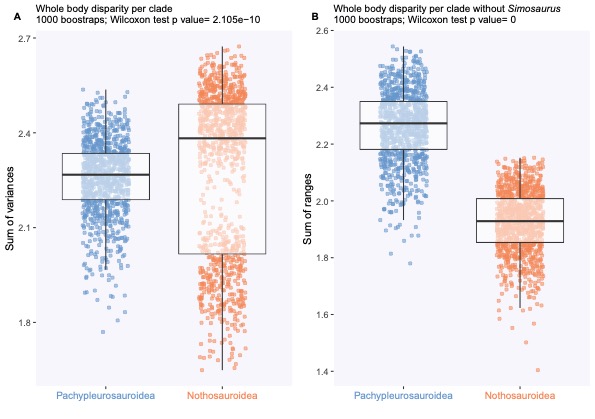
**

**Supplementary figure 14:** Whole body disparity distribution per clade (A) and with *Simosaurus gaillardoti* removed (B)

**
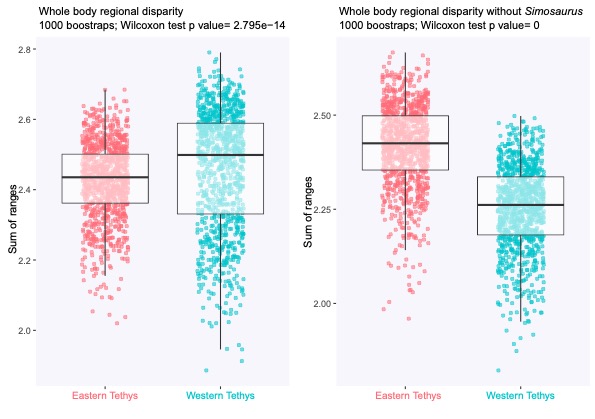
**

**Supplementary figure 15:** Whole body regional disparity distribution with all species of the dataset (A) and with *Simosaurus gaillardoti* removed (B)

**
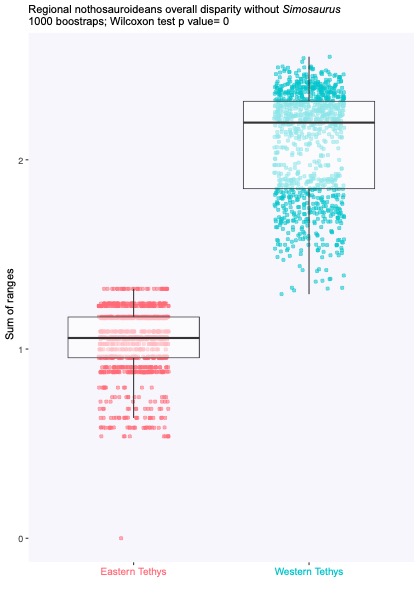
**

**Supplementary figure 16:** Whole body regional nothosauroidean disparity distribution with *Simosaurus gaillardoti* removed (B)

Supplementary references

Anderson P. 2009. Biomechanics, functional patterns, and disparity in Late Devonian arthrodires. *Paleobiology* 35:321–342. DOI: 10.1666/0094-8373-35.3.321.

Anderson PSL, Friedman M, Brazeau MD, Rayfield EJ. 2011. Initial radiation of jaws demonstrated stability despite faunal and environmental change. *Nature* 476:206–209. DOI: 10.1038/nature10207.

Bennion RF, MacLaren JA, Coombs EJ, Marx FG, Lambert O, Fischer V. 2022. Convergence and constraint in the cranial evolution of mosasaurid reptiles and early cetaceans. *Paleobiology*:1–17. DOI: 10.1017/pab.2022.27.

Busbey A. 1995. The structural consequences of skull flattening in crocodilians. In: Thomason J ed. *Functional morphology in vertebrate paleontology.* Cambridge University Press, 173–192.

Carroll RL, Gaskill P, Whittington HB. 1985. The nothosaur *Pachypleurosaurus* and the origin of plesiosaurs. *Philosophical Transactions of the Royal Society of London. B, Biological Sciences* 309:343–393. DOI: 10.1098/rstb.1985.0091.

Fischer V, Bennion RF, Foffa D, MacLaren JA, McCurry MR, Melstrom KM, Bardet N. 2022. Ecological signal in the size and shape of marine amniote teeth. *Proceedings of the Royal Society B: Biological Sciences* 289:20221214. DOI: 10.1098/rspb.2022.1214.

Fischer V, Benson RBJ, Zverkov NG, Soul LC, Arkhangelsky MS, Lambert O, Stenshin IM, Uspensky GN, Druckenmiller PS. 2017. Plasticity and convergence in the evolution of short-necked plesiosaurs. *Current Biology* 27:1667–1676. DOI: 10.1016/j.cub.2017.04.052.

Fischer V, Maclaren JA, Bennion RF, Druckenmiller PS, Benson RBJ. 2020. The macroevolutionary landscape of short-necked plesiosaurians. *Scientific Reports*.

Foffa D, Young MT, Stubbs TL, Dexter KG, Brusatte SL. 2018. The long-term ecology and evolution of marine reptiles in a Jurassic seaway. *Nature Ecology & Evolution* 2:1548–1555. DOI: 10.1038/s41559-018-0656-6.

Großmann F. 2007. The taxonomic and phylogenetic position of the Plesiosauroidea from the Lower Jurassic Posidonia shale of South-West Germany. *Palaeontology* 50:545–564. DOI: <https://doi.org/10.1111/j.1475-4983.2007.00654.x>.

Hinić-Frlog S, Motani R. 2010. Relationship between osteology and aquatic locomotion in birds: determining modes of locomotion in extinct Ornithurae. *Journal of Evolutionary Biology* 23:372–385. DOI: https://doi.org/10.1111/j.1420-9101.2009.01909.x.

Holzman R, Collar DC, Price SA, Hulsey CD, Thomson RC, Wainwright PC. 2012. Biomechanical trade-offs bias rates of evolution in the feeding apparatus of fishes. *Proceedings of the Royal Society B: Biological Sciences* 279:1287–1292. DOI: 10.1098/rspb.2011.1838.

Krahl A. 2021. The locomotory apparatus and paraxial swimming in fossil and living marine reptiles: comparing Nothosauroidea, Plesiosauria, and Chelonioidea. *PalZ* 95:483–501. DOI: 10.1007/s12542-021-00563-w.

MacLaren JA, Anderson PSL, Barrett PM, Rayfield EJ. 2017. Herbivorous dinosaur jaw disparity and its relationship to extrinsic evolutionary drivers. *Paleobiology* 43:15–33. DOI: 10.1017/pab.2016.31.

MacLaren JA, Bennion RF, Bardet N, Fischer V. 2022. Global ecomorphological restructuring of dominant marine reptiles prior to the Cretaceous–Palaeogene mass extinction. *Proceedings of the Royal Society B: Biological Sciences* 289:20220585. DOI: 10.1098/rspb.2022.0585.

Massare JA. 1987. Tooth morphology and prey preference of Mesozoic marine reptiles. *Journal of Vertebrate Paleontology* 7:121–137. DOI: 10.1080/02724634.1987.10011647.

Massare JA. 1988. Swimming capabilities of Mesozoic marine reptiles: implications for the methods of predation. *Palaeobiology* 14:187–205.

Mccurry MR, Fitzgerald EMG, Evans AR, Adams JW, Mchenry CR. 2017. Skull shape reflects prey size niche in toothed whales. *Biological Journal of the Linnean Society* In Press:1–11.

Neenan JM, Klein N, Scheyer TM. 2013. European origin of placodont marine reptiles and the evolution of crushing dentition in Placodontia. *Nature Communications* 4:1621.

O’Keefe FR. 2001. A cladistic analysis and taxonomic revision of the Plesiosauria (Reptilia:Sauropterygia). *Acta Zoological Fennica* 213:1–63.

Ridgway SH, Harrison R. 1999. *The Second Book of Dolphins and the Porpoise. Handbook of Marine Mammals*. Cambridge: Academic Press.

Rieppel O. 2002. Feeding mechanics in Triassic stem-group sauropterygians: the anatomy of a successful invasion of Mesozoic seas. *Zoological Journal of the Linnean Society* 135:33–63.

Stayton CT. 2006. Testing hypotheses of convergence with multivariate data: Morphological and functional convergence among herbivorous lizards. *Evolution* 60:824–841. DOI: https://doi.org/10.1111/j.0014-3820.2006.tb01160.x.

Stubbs TL, Benton MJ. 2016. Ecomorphological diversifications of Mesozoic marine reptiles: the roles of ecological opportunity and extinction. *Paleobiology*:1–27. DOI: 10.1017/pab.2016.15.

Sues H-D. 1987. Postcranial skeleton of *Pistosaurus* and interrelationships of the Sauropterygia (Diapsida). *Zoological Journal of the Linnean Society* 90:109–131. DOI: 10.1111/j.1096-3642.1987.tb01351.x.

Taylor MA. 1987. How tetrapods feed in the water: a functional analysis by paradigm. *Zoological Journal of the Linnean Society* 91:171–195.

Xu G-H, Ren Y, Zhao L-J, Liao J-L, Feng D-H. 2022. A long-tailed marine reptile from China provides new insights into the Middle Triassic pachypleurosaur radiation. *Sicentific reports* 12. DOI: 10.1038/s41598-022-11309-2.

Zeffer A, Johansson LC, Marmebro Å. 2003. Functional correlation between habitat use and leg morphology in birds (Aves). *Biological Journal of the Linnean Society* 79:461–484. DOI: 10.1046/j.1095-8312.2003.00200.x.

Zverkov NG, Fischer V, Madzia D, Benson RBJ. 2018. Increased pliosaurid dental disparity across the Jurassic – Cretaceous transition. *Palaeontology* In Press:1–22. DOI: 10.1111/pala.12367.
