# Supplementary material for: High phenotypic plasticity at the dawn of the eosauropterygian radiation
Source: PeerJ. 2023 Sep 1;11:e15776. doi: 10.7717/peerj.15776 (PMC10476616; doi:10.7717/peerj.15776)
Supplement: Supplemental Information 3 [file peerj-11-15776-s003.pdf]

# High phenotypic plasticity at the dawn of the eosauropterygian radiation: Script for analyses

2023-05

## 1. Package installation and loadings

### 1.1 Installation of packages

```
library(openxlsx)
library(dplyr)
library(ggplot2)
library(ggthemes)
library(ggrepel)
library(dplyr)
library(cluster)
library(ape)
library(vegan)
library(egg)
library(psych)
library(pvclust)
library(ggdendro)
library(paleotree)
library(strap)
library(paleotree)
library(phytools)
library(convevol)
library(viridis)
library(dispRity)
library(dendextend)
library(phylogram)
library(adephylo)
library(HH)
library(phylobase)
library(dplyr)
library(RRphylo)
```

## 2. Importation of the data

### 2.1. Importation and edition of the morphological dataset

The data are imported from a .csv file generated in excel.

```
morpho_full<- read_xlsx("Data_disparity_Triassic_eosauropterygians.xlsx",
sheet = "Species_traits", colNames=TRUE)
```

Change the row names with the name of the species present in the dataset; also stock the names of the species and their corresponding clades in other variables called 'Species' and 'Clade' respectively.

```
rownames(morpho_full) <- morpho_full$Species
Species <- morpho_full$Species
morpho_full<- morpho_full[,-2]
Clade <- as.character(morpho_full$Clade)
```

## 2.2. Importation of proxy of the size of the species.

These data are imported from a .csv file. For the whole-body and craniodental analyses, the size of the skull is used while the length of the humerus is used for the postcranial analyses.

```
Skull_size_to_plot <-
read_xlsx("Data_disparity_Triassic_eosauropterygians.xlsx", sheet =
"Skull_size_to_plot", colNames=TRUE)
rownames(Skull_size_to_plot) <- Skull_size_to_plot$Species;
Skull_size_to_plot <- Skull_size_to_plot[,-2]
log_skull_size_to_plot <- log(Skull_size_to_plot$Skull.size)

Posterior_skull_width <-
read_xlsx("Data_disparity_Triassic_eosauropterygians.xlsx", sheet =
"posterior_skull_width_to_plot", colNames=TRUE)
rownames(Posterior_skull_width)=posterior_skull_width$Species;
posterior_skull_width <- posterior_skull_width[,-2]

Humerus_size_to_plot <-
read_xlsx("Data_disparity_Triassic_eosauropterygians.xlsx", sheet =
"Humerus_size_to_plot", colNames=TRUE)
rownames(Humerus_size_to_plot) <- Humerus_size_to_plot$Species;
Humerus_size_to_plot <- Humerus_size_to_plot[,-2]
log_humerus_size_to_plot <- log(Humerus_size_to_plot$Humerus.length)
```

## 2.3. Importation of the location of each species

```
eosauropt_location <-
read_xlsx("Data_disparity_Triassic_eosauropterygians.xlsx", sheet =
"Eosauropt_location", colNames=TRUE)
rownames(eosauropt_location) <- eosauropt_location$Species
eosauropt_location <- eosauropt_location[-2]
location <- eosauropt_location$Location
```

## 2.4. Importation of the age of the species

The second file contains the stage of each species and the second the FAD and LAD of each stages. The reason justifying the presence of two different files is that some species are present in more than one stage and thus have been duplicated in the second. This will be serve in the section 7.2 to visualize changes in the morphospace occupation through time.

```
eosauropt_timescaling <-
read_xlsx("Data_disparity_Triassic_eosauropterygians.xlsx", sheet =
"Eosauropt_timescaling", colNames=TRUE)
rownames(eosauropt_timescaling) <- eosauropt_timescaling$Species
eosauropt_timescaling<- eosauropt_timescaling[,-2]

eosauropt_age <-read_xlsx("Data_disparity_Triassic_eosauropterygians.xlsx",
sheet = "Eosauropt_age", colNames=TRUE)
rownames(eosauropt_age) <- eosauropt_age$Species; eosauropt_age <-
eosauropt_age[-2]
Age<-eosauropt_age$Stage
```

### 2.5. Assigning colours to clades

Specify the colour of each clade according the alphabetic order. Thus, the first color will be attributed to the nothosauroids, the second to the pachypleurosauroids and the last one to the pistosauroids.

```
clade_colours<- c('#F48656','#6697cc','#5b2977')
```

### 2.6 Showing the amount of missing data

```
paste("Amount of missing
data:",100*(sum(is.na(morpho_full))/(dim(morpho_full)[1]*dim(morpho_full)[2])
),"%")

paste("Amount of craniodental missing
data:",100*(sum(is.na(morpho_full[,2:22]))/(dim(morpho_full[,2:22])[1]*dim(mo
rpho_full[,2:22])[2])),"%")

paste("Amount of postcranial missing
data:",100*(sum(is.na(morpho_full[,23:32]))/(dim(morpho_full[,23:32])[1]*dim(
morpho_full[,23:32])[2])),"%")
```

## 3. Division of the dataset according the distinct body region

### 3.1 Craniodental data

Select the quantitative and discrete traits that are related to the craniodental region.

```
craniodental_dataset <- morpho_full[,1:23]
```

#### 3.1.1. Keeping species that passed the threshold of 40% of completeness

```
threshold <- 0.40
craniodental_completeness <-
rowSums(!is.na(craniodental_dataset))/ncol(craniodental_dataset)
paste("craniodental completeness", Species, craniodental_completeness*100,
"%") # Showing the  craniodental completeness of each species
craniodental_dataset_tres <-
craniodental_dataset[craniodental_completeness>threshold,] #Keeping species
```

```
that passed the treshold
craniodental_dataset_tres <- cbind (Clade,craniodental_dataset_tres)
```

#### 3.1.2. Exploration of the correlation between quantitative craniodental traits

Discrete features characterizing the morphology of the snout or the teeth are not taken into account in this correlation.

```
pairs.panels(craniodental_dataset_tres[,2:19],method="pearson",lm=TRUE,hist.c
ol="grey",bg=c('#f7b055','#6697cc','#5b2977') [morpho_full$Clade], pch =
c(21,22,24)
[craniodental_dataset_tres$Clade],density=FALSE,ellipses=FALSE,stars=TRUE)
```

### 3.2. Postcranial data

We need to select the quantitative and discrete traits that are related to the postcranial region.

```
postcranial_dataset <- morpho_full[,24:32]
postcranial_dataset<- cbind(Clade,postcranial_dataset)
```

#### 3.2.1. Keeping species that passed the threshold of 40% of completeness in a new dataset

```
threshold <- 0.39
completeness <- rowSums(!is.na(postcranial_dataset[,-
1]))/ncol(postcranial_dataset[,-1])
paste("Postcranial completeness", Species, completeness*100, "%")
postcranial_dataset_tres<- postcranial_dataset[completeness>threshold,]
#Keeping species that passed the treshold
Postcranial_species <- rownames(postcranial_dataset_tres)
Clade2 <- postcranial_dataset_tres$Clade #Keeping the name of the clades of
species that passed the treshold
Clade2 <- as.character(Clade2) #Converting the clade as characters.
```

#### 3.2.2. Exploration of the correlation between quantitative postcranial traits

```
pairs.panels(postcranial_dataset_tres[,-1],method="pearson",lm=TRUE,
hist.col="grey",bg=c('#f7b055','#6697cc','#5b2977')
[postcranial_dataset_tres$Clade], pch = c(21,22,24)
[postcranial_dataset_tres$Clade], density=FALSE,ellipses=FALSE,stars=TRUE)
```

### 3.3. Whole body data

#### 3.3.1. Keeping species that passed the treshold of 40% of completeness in a new dataset

```
threshold <- 0.40
completeness <- rowSums(!is.na(morpho_full))/ncol(morpho_full) #Keeping
species that passed the treshold
paste("Whole body completeness",Species, completeness*100, "%")
morpho_full_tres <- morpho_full[completeness>threshold,]
```

## 4. Scaling the dataset and computing the distance matrix

**In the rest of this document, only the lines of code concerning the analyses performed with whole-body dataset will be displayed to show how major analyses have been realized.** The procedure whould be the same for the craniodental and postcranial exclusive dataset by using the data sets produced beforehand in this document.

### 4.1. Scaling only continuous variables of the dataset.

```
morpho_full_tres[2:18] <- scale(morpho_full_tres[,2:18]);
morpho_full_tres[,24:32] <- scale(morpho_full_tres[,24:32])
```

### 4.2. Computing the distance matrix

As the whole-body dataset contains continuous but also discrete traits, the Euclidean method is inapplicable and using the gower distance metric is more accurate.

```
whole_body_dissimilarity_matrix <- daisy(morpho_full_tres[,2:32], metric=
"gower" )#Daisy function and gower metric because of the discrete variables
whole_body_dissimilarity_matrix <- as.matrix(whole_body_dissimilarity_matrix)
```

## 5. Cluster dendrogram analysis

### 5.1. Compute the hierarchical cluster analysis

```
whole_body_clust_result <-
pvclust(as.data.frame(t(whole_body_dissimilarity_matrix)),
method.hclust="ward.D2", use.cor="pairwise.complete.obs", nboot=1000,
r=seq(0.5,10,by=.5))
```

### 5.2. Prepare data to compute the dendrogram and plot it

```
whole_body_dendro <-
dendro_data(whole_body_clust_result$hclust,type="rectangle")
clade_df <- as.data.frame(Clade) ; rownames(clade_df) <-
rownames(morpho_full) ; clade_df$label <- rownames(morpho_full_tres)
whole_body_dendro$labels <- merge(whole_body_dendro$labels, clade_df,
by="label")

whole_body_cluster <- ggplot()+
  geom_segment(data=segment(whole_body_dendro),aes(x=x, y=y, xend=xend,
yend=yend))+
  geom_text(data=label(whole_body_dendro), aes(x, y, label=label,color
=Clade, fontface=3, hjust=0, size=3)) +
  labs(y="Height",x="")+
  scale_color_manual(values = clade_colours)+
  expand_limits(y=c(1.1*max(whole_body_clust_result$hclust$height),-
0.4*max(whole_body_clust_result$hclust$height)))+
  scale_x_continuous(breaks=NULL)+
  coord_flip() +
```

```
scale_y_reverse(breaks=c(max(whole_body_dendro$segment$yend),0),minor_breaks=
NULL)
```

# 6. Morphospace and phylomorphospace occupation analyses

## 6.1. Phylogenetic data

### 6.1.1. Importation of phylogenetic trees

As the matrix of Xu et al., 2022 does not contains all the taxa present in the morphological dataset, we have to add manually six taxa according the literature. References for the phylogenetic position of each of these six taxa have been added following their corresponding code line.

```
eosauropt_tree <- read.nexus("eosauropt_trees_IW12.nex")
eosauropt_tree <- bind.tip(eosauropt_tree, "Neusticosaurus_peyeri",
edge.length=NULL,
where=which(eosauropt_tree$tip.label=="Neusticosaurus_pusillus"), position=0)
#as sister lineage of N. pusillus based on Klein et al., 2022
eosauropt_tree <- bind.tip(eosauropt_tree, "Neusticosaurus_edwardsii",
edge.length=NULL,
where=which(eosauropt_tree$tip.label=="Neusticosaurus_peyeri"), position=0)
#as sister lineage of N. peyeri based on Klein et al., 2022
eosauropt_tree <- bind.tip(eosauropt_tree, "Prosantosaurus_scheffoldi",
edge.length=NULL, where =67, position =0) #as sister lineage of the clade
Neusticosaurus - Serpianosaurus based on Klein et al., 2022
eosauropt_tree <- bind.tip(eosauropt_tree,
"Brevicaudosaurus_jiyangshanensis", edge.length = NULL, where =80,
position=0) #as sister lineage of the Nothosauridae according Shang et al.,
2020
eosauropt_tree <- bind.tip(eosauropt_tree, "Nothosaurus_luopingensis",
edge.length=NULL,
where=which(eosauropt_tree$tip.label=="Nothosaurus_yangjuanensis"),
position=0) # as sister lineage of Nothosaurus yangiuanensis according Shang
et al., 2022
eosauropt_tree <- bind.tip(eosauropt_tree, "Luopingosaurus_imparilis",
edge.length = NULL,
where=which(eosauropt_tree$tip.label=="Honghesaurus_longicaudalis"),
position=0) #as sister lineage of Dianopachysaurus according Chang et al.,
2022
```

### 6.1.2. Only keep the species that are present in the morphological dataset

Verify if all taxa of the morphological dataset are present in our phylogenetic tree.

```
whole_body_tree <-
drop.tip(eosauropt_tree,eosauropt_tree$tip.label[!eosauropt_tree$tip.label
%in% rownames(morpho_full_tres)])
```

```
rownames(morpho_full_tres)%in% whole_body_tree$tip.label # which taxa with morphological data are not present in the phylogenetic tree?
```

### 6.1.3. Timescaling

In this section, use the dataset **eosauropt_timescaling** which ahve been previously imported. Tree are calibrated with the minimum branch length algorithm using a minimum value 0.5 Myr. The timescaled phylogenetic tree is plotted with the international geological time scale.

```
whole_body_tree_mbl <-
timePaleoPhy(whole_body_tree,timeData=eosauropt_timescaling[,3:4],type=
"mbl",vartime=0.5,dateTreatment="firstLast")

geoscalePhylo(ladderize(whole_body_tree_mbl,right=TRUE),eosauropt_timescaling
[,3:4],cex.ts=0.8,cex.tip=0.7, x.lim = c(230,260))
```

## 6.2. PCoA analysis

### 6.2.1. Computing the PCoA

```
pcoa_whole_body <-
pcoa(whole_body_dissimilarity_matrix,correction="cailliez")
```

### 6.2.2. Prepare data for plotting the morphospace occupation generated with the PCoA

**df_pcoa_whole_body** is a dataframe containing the PCoA scores on the axes 1 and 2 for each species.

```
df_pcoa_whole_body<- cbind (as.data.frame(pcoa_whole_body$vectors[,1:2]),
Clade)

ecomorpho_split_ratios <- split(df_pcoa_whole_body, df_pcoa_whole_body$Clade)
#Create a convex hull to plot on the ggplot
chull_pcoa_whole_body_ratios <- lapply (ecomorpho_split_ratios, function(df)
{
  df[chull(df),] # chull computes convex hulls for each subgroup.
})
chull_pcoa_whole_body_ratios <- do.call (rbind,chull_pcoa_whole_body_ratios )
#combines the coordinates of the points (i.e. taxa) needed to draw the convex
hull (i.e. only the extremes ones)

plot_pcoa_whole_body_ratios <- ggplot (data=df_pcoa_whole_body, aes
(x=Axis.1, y=Axis.2, colour=Clade)) +
  theme_minimal() +
  geom_point(aes(color=Clade,size=3, alpha=1, shape=location)) +
  geom_rangeframe(color = "black") +
  geom_text_repel(aes(label=rownames(morpho_full), fontface=3), size=4)+
  scale_color_manual(values = clade_colours)+
  scale_fill_manual(values=clade_colours)+
   theme_minimal()+
  theme(panel.background =
```

```
element_rect(fill="#FBF9FB"),legend.position="none",panel.grid.minor=element_
blank())
  labs(title= "Triassic eosauropterygian ecomorphospace", x = paste0('Axis 1
= ', round(((pcoa_whole_body$values$Rel_corr_eig[1])*100), digits = 2), '%'),
       y = paste0('Axis 2 = ',
round(((pcoa_whole_body$values$Rel_corr_eig[2])*100), digits = 2), '%')) +
  geom_polygon(data=chull_pcoa_whole_body_ratios, aes(x=Axis.1, y=Axis.2,
fill=Clade), alpha=1/3,color="NA")
```

Before computing the phylomorphospace superimposed on the density of taxa, load the function **ggphylomorpho** from [Fischer *et al.* (2020)] (https://www.nature.com/articles/s41598-020-73413-5).

```
source('GGphylomorpho_density.R', chdir = TRUE)
```

## 6.3. Phylomorphospace generated with the PCoA

Prepare the tipinfo argument in the function ggphylomorpho by adding the name of the species in an additional column

```
df_pcoa_whole_body_ratios <- cbind(as.data.frame(df_pcoa_whole_body),
Species)
```

### 6.3.1. Computation of the phylomorphospace using the first 2 axes of the PCoA

Use the scores on the first 2 axes of the PCoA to generate the density of taxa by using **bandw.**

```
df_pcoa_whole_body[,1:2] <-10*df_pcoa_whole_body[,1:2] # Better visualization
of the density
df_pcoa_whole_body$location <- location
bandw <-c(max(df_pcoa_whole_body[,1]),max(df_pcoa_whole_body[,2]))

Whole_body_phylo_MS_pcoa <- ggphylomorpho(tree= whole_body_tree_mbl, tipinfo
=df_pcoa_whole_body, names=TRUE, xvar=Axis.1, yvar=Axis.2,factorvar= Clade,
labelvar = Species, tree.alpha = 0.7, repel = TRUE, h=bandw, sizedata =
log_skull_size_to_plot)+

coord_fixed(ratio=1,xlim=c(min(df_pcoa_whole_body[,1])*1.3,max(df_pcoa_whole_
body[,1])*1.3),ylim=c(min(df_pcoa_whole_body[,2])*1.6,max(df_pcoa_whole_body[
,2])*1.1))+
  scale_color_manual(values = clade_colours)+
  scale_fill_viridis(option="viridis")+
  #scale_fill_manual(values=clade_colours)+
  theme_minimal()+
  theme(panel.background =
element_rect(fill="#FBF9FB"),legend.position="none",panel.grid.minor=element_
blank())
Whole_body_phylo_MS_pcoa <- Whole_body_phylo_MS_pcoa+ labs(title=
paste("Phylomorphospace occupation (PCoA) \nwhole body dataset"),
x=paste('Axis 1 = ', round(((pcoa_whole_body$values$Rel_corr_eig[1])*100),
```

```
digits = 2), '%'), y= paste('Axis 2 = ',
round(((pcoa_whole_body$values$Rel_corr_eig[2])*100), digits = 2), '%'))
Whole_body_phylo_MS_pcoa
```

## 6.4. nMDS analysis

### Compute the nMDS and the stressplot

The nMDS is generated with 2 dimensions. The stressplot shows the relationship between the dissimilarities among taxa present in the dissimilarity matrix and the ordination on the nMDS morphospace (maximum number of random starts = 100).

```
nmds_whole_body <- metaMDS(whole_body_dissimilarity_matrix,k=2,trymax=100)
stressplot(nmds_whole_body) ## sees how the models fit.
```

### 6.4.1. prepare data for plotting the morphospace occupation generated with the nMDS

**df_nmds_whole_body** is a dataframe containing the scores of the Nonmetric Multidimensional Scaling (nMDS) generated with 2 dimensions for each species.

```
df_nmds_whole_body <- as.data.frame(nmds_whole_body$points) ;
df_nmds_whole_body$Species <- rownames(morpho_full); df_nmds_whole_body$Clade
<- Clade

split(df_nmds_whole_body,df_nmds_whole_body$Clade) #Create a convex hull to
plot on the ggplot
chull_nmds_whole_body <- lapply(split(df_nmds_whole_body,
df_nmds_whole_body$Clade), function(df){
  df[chull(df),]
})
chull_nmds_whole_body <- do.call(rbind, chull_nmds_whole_body) #combines the
coordinates of the points (i.e. taxa) needed to draw the convex hull (i.e.
only the extremes ones)

plot_NMDS_whole_body <-  ggplot(data=df_nmds_whole_body, aes(x=MDS1,y=MDS2,
colour=Clade)) +
  geom_point(aes(color=Clade,size= 3,alpha=1, shape=location)) +
  geom_rangeframe(color = "black") +
  scale_color_manual(values = clade_colours)+
  scale_fill_manual(values=clade_colours)+
  theme(panel.background = element_rect(fill="#d8d0f032"))+
  theme(panel.grid.major = element_blank(), panel.grid.minor =
element_blank())+
  geom_text_repel(aes(label=Species), size=3) +
  labs( title= "Triassic eosauropterygian nMDS ecomorphospace", x = "nMDS 1",
      y = "nMDS 2") +
  geom_polygon(data=chull_nmds_whole_body, aes(x=MDS1,y=MDS2, fill=Clade),
alpha=0.3,color="NA")
```

### 6.5. Phylomorphospace generated with the nMDS

You need to prepare the tipinfo argument in the function ggphylomorpho by adding the name of the species in an additional column

```
df_nmds_whole_body <- cbind(as.data.frame(df_nmds_whole_body), Species) #
Whole body region
```

#### 6.5.1. Computation of the phylomorphospace using the first 2 dimensions of the nMDS

```
df_nmds_whole_body[,1:2] <- 10*df_nmds_whole_body[,1:2]
bandw <-c(max(df_nmds_whole_body[,1]),max(df_nmds_whole_body[,2]))
df_nmds_whole_body$location <- location
df_nmds_whole_body$location <- as.character(df_nmds_whole_body$location)
Whole_body_phylo_MS_NMDS <- ggphylomorpho(tree= whole_body_tree_mbl, tipinfo
=df_nmds_whole_body, names=TRUE, xvar=MDS1, yvar=MDS2,factorvar= Clade,
labelvar = Species, pointshape=df_nmds_whole_body$location, tree.alpha = 0.7,
repel = TRUE, h=bandw, sizedata = log_skull_size_to_plot)+

coord_fixed(ratio=1,xlim=c(min(df_nmds_whole_body[,1]),max(df_nmds_whole_body
[,1])*1.1),ylim=c(min(df_nmds_whole_body[,2])*1.1,max(df_nmds_whole_body[,2])
*1.1))+
  scale_color_manual(values = clade_colours)+
  scale_fill_manual(values=clade_colours)+
  theme_minimal()+
  theme(panel.background =
element_rect(fill="#FBF9FB"),legend.position="none",panel.grid.minor=element_
blank())
Whole_body_phylo_MS_NMDS <- Whole_body_phylo_MS_NMDS+ labs(title=
paste("Phylomorphospace occupation (NMDS) \nwhole body dataset"),
x=paste('NMDS1'), y= paste('NMDS2 '))
```

## 7. Morphospace occupation for Western and Eastern Tethys during the Middle Triassic

In this section, the morphospace occupation are computed through the Middle Triassic for the Western and Eastern Tethys respectively by using the scores on the first two axes of the PCoA. The density displayed in the morphospace is the one that has been computed with the complete dataset in the section 6.3.1. Here is an example of the display of morphospace occupation of Western Tethys species during the overall Middle Tethys and also during the Bithynian (early Middle Triassic, Anisian). Computing the same analyses for an other time bin just require to change the name of the substage and analyses for the Eastern Tethys follow the same protocol.

### 7.1. Preparing dataset

Here, scores and information for species that are present on more than one substage need to be duplicated

```
PCoA_whole_body_data <- df_pcoa_whole_body %>% slice(1,rep(2,3),3:27,rep
(28,4),29,rep(30,3),rep(31,2),32, rep(33,2),34:36);
row.names(PCoA_whole_body_data)[2] <- "Anarosaurus_heterodontus_Bithy";
row.names(PCoA_whole_body_data)[3] <- "Anarosaurus_heterodontus_Pelso";
row.names(PCoA_whole_body_data)[4] <- "Anarosaurus_heterodontus_Illy"
row.names(PCoA_whole_body_data)[30] <- "Nothosaurus_giganteus_Ill";
row.names(PCoA_whole_body_data)[31] <-
"Nothosaurus_giganteus_Fassa";row.names(PCoA_whole_body_data)[32] <-
"Nothosaurus_giganteus_Longo"; row.names(PCoA_whole_body_data)[33] <-
"Nothosaurus_giganteus_Julia";
row.names(PCoA_whole_body_data)[35] <-
"Nothosaurus_marchicus_Bithy";row.names(PCoA_whole_body_data)[36] <-
"Nothosaurus_marchicus_Pelso";row.names(PCoA_whole_body_data)[37] <-
"Nothosaurus_marchicus_Illy";
row.names(PCoA_whole_body_data)[38] <- "Nothosaurus_mirabilis_Bithy";
row.names(PCoA_whole_body_data)[39] <- "Nothosaurus_mirabilis_Fassa"
row.names(PCoA_whole_body_data)[41] <- "Simosaurus_gaillardoti_Fassa";
row.names(PCoA_whole_body_data)[42] <- "Simosaurus_gaillardoti_Longo";

PCoA_whole_body_data <- cbind (PCoA_whole_body_data, Age) #add age of the
species; see section 2.3
PCoA_whole_body_data <- cbind(PCoA_whole_body_data,location_age) # add the
location of each row of the dataset
```

This dataset is splitted according the location (Western and Eastern Tethys respectively).

```
PCoA_all_bins <- split(PCoA_whole_body_data,
PCoA_whole_body_data$location_age)
```

## 7.2 Analyses for the whole Middle Triassic and the for Bithyinan in the Western Tethys

### 7.2.1 Whole Middle Triassic

```
PCoA_all_bins_WT_age <- split(PCoA_all_bins$Western_Tethys,
PCoA_all_bins$Western_Tethys$Age) #Need to replace Western_Tethys by
Easter_Tethys if you want to visualize the morphospace occupation of the
other tethyan region

plot_PCoA_WT_all_bins<- ggplot(data=PCoA_all_bins$Western_Tethys,
aes(x=Axis.1,y=Axis.2)) +
  stat_density_2d(data = df_pcoa_whole_body,aes(x =Axis.1, y =Axis.2,
alpha=(..level..)^12),h=bandw,geom="polygon")+
  scale_alpha(guide="none")+
  scale_fill_gradient(low = "gray", high = "darkgrey") +
  geom_point(aes(color=Clade,size= 10,alpha=1, stroke=0.4)) +
  geom_rangeframe(color = "black") +
  scale_color_manual(values = clade_colours)+
  scale_fill_manual(values=clade_colours)+
  scale_x_continuous(limits=c(-4,4))+
  scale_y_continuous(limits=c(-3,3))+
```

```
  theme(plot.title=element_text(size = 15,
colour="#00c6cc"),axis.text=element_text(size=8),axis.title=element_text(size
=10),legend.position = "none",panel.background =
element_rect(fill="#d8d0f032"),panel.grid.major = element_blank(),
panel.grid.minor = element_blank())+
  labs(title="Western Tethys - all bins",x=paste('Axis 1 = ',
round(((pcoa_whole_body$values$Rel_corr_eig[1])*100), digits = 2), '%'), y=
paste('Axis 2 = ', round(((pcoa_whole_body$values$Rel_corr_eig[2])*100),
digits = 2), '%'))
plot_PCoA_WT_all_bins
```

### 7.2.2. Bithynian in the Western Tethys

```
plot_PCoA_WT_Bithynian<- ggplot(data=PCoA_all_bins_WT_age$Bithynian,
aes(x=Axis.1,y=Axis.2)) + #Need to replace the name of the substage if you
want to visualize the morphospace occupation in an other substage of the
Middle Triassic in the Western Tethys.
  stat_density_2d(data = df_pcoa_whole_body,aes(x =Axis.1, y =Axis.2,
alpha=(..level..)),h=bandw,geom="polygon")+
  scale_alpha(guide="none")+
  scale_fill_gradient(low = "gray", high = "darkgrey") +
  geom_point(aes(color=Clade,size= 6,alpha=1, stroke=0.4)) +
  geom_rangeframe(color = "black") +
  scale_color_manual(values = clade_colours)+
  scale_fill_manual(values=clade_colours)+
  scale_x_continuous(limits=c(-4,4))+
  scale_y_continuous(limits=c(-3,3))+
  theme(plot.title=element_text(size = 15,
colour="#00c6cc"),axis.text=element_text(size=8),axis.title=element_text(size
=10),legend.position = "none",panel.background =
element_rect(fill="#d8d0f032"),panel.grid.major = element_blank(),
panel.grid.minor = element_blank())+
  labs(title="Bithynian",x=paste('Axis 1 = ',
round(((pcoa_whole_body$values$Rel_corr_eig[1])*100), digits = 2), '%'), y=
paste('Axis 2 = ', round(((pcoa_whole_body$values$Rel_corr_eig[2])*100),
digits = 2), '%'))
plot_PCoA_WT_Bithynian
```

NB If you want to combine all the morphospace occupation for both Western and Eastern Tethys as in Fig.3 (D-L):

```
plot<-ggarrange(plot_PCoA_WT_all_bins,plot_PCoA_WT_Bithynian,
plot_PCoA_WT_Pelsonian,plot_PCoA_WT_Illyrian, plot_PCoA_WT_Fassanian,
plot_PCoA_WT_Longobardian, plot_PCoA_ET_all_bins, plot_PCoA_ET_Pelsonian,
plot_PCoA_ET_Longobardian, ncol=3, nrow=3,widths = c(2,2,2), heights =
c(2,2,2), labels=c("D","E","F","G","H","I","J","K","L"))
```

# 8.Tanglegram and Mantel Test

## 8.1. Tanglegram

For generating a tanglegram, the first step is to transfom our phylogenetic tree (**whole_body_tree_mbl**) in an ultrametric tree (all tips are equidistant from the root) with our previoulsy .

```
whole_body_dendro_phylo<- as.dendrogram(force.ultrametric(tree =
ladderize(whole_body_tree_mbl), method="extend"))
```

The next step is to extract cluster data from the cluster dendrogram analyses generated with the **pvclust** function.

```
whole_body_cluster <- as.dendrogram(whole_body_clust_result$hclust) #extract
cluster from pvclust result
```

### Computing the tanglegram

```
par(mfrow=c(2,1))
gg <-tanglegram(whole_body_dendro_phylo_2,whole_body_cluster, fast = TRUE,
margin_inner = 12,main_left="Phylogenetic tree",main_right="Cluster
dendrogram",axes=FALSE,cex_main=1.5) #creates tanglegram
```

## 8.2. Mantel test

The first step here is to define a distance matrix using our timescaled phylogenetic tree.

Compute the the phylogenetic distances between tips.

```
phylo_dist_mbl <- distTips(whole_body_tree_mbl,method="nNodes")
```

Finally, we need to run the mantel test.

```
whole_body_dissimilarity_matrix <-dist(whole_body_dissimilarity_matrix)
mantel_phenoVSphylo_mb <-
mantel.rtest(phylo_dist_mbl,whole_body_dissimilarity_matrix, nrepet=1000)
```

# 9. Computing total disparity

In the per-clade analyses, only total disparity of Pachypleurosauroidea and Nothosauroidea are computed, given the small number of pistosauroids. However, they are sampled in the Tethyan regional disparities analyses with the exception of *Augustasaurus hagdorni* which have been found in the East Panthalassa.

## 9.1. Disparity Pachypleurosauroidea vs Nothosauroidea

Firstly, set the number of boostraps.

```
number_bootstraps <- 1000
```

Extract the scores on all axes of the PCoA for each taxon and remove the pistosauroids.

```
pcoa_whole_body_vectors <- as.data.frame(pcoa_whole_body$vectors[-(34:36),])
# remove the three pistosauroids. If you want to investigate the disparity of
nothosaurians (without Simosaurus), select the lines 33 to 36
pcoa_whole_body_vectors$Clade <- Clade[-(34:36)]
```

Create subsets (split) of the dataset according the clade of the species.

```
whole_body_disp_clades <- lapply(split(pcoa_whole_body_vectors,
pcoa_whole_body_vectors$Clade), rownames)
subsets_whole_body_clades <-
custom.subsets(data=data.matrix(pcoa_whole_body$vectors),group=whole_body_dis
p_clades)
```

Bootstrap each subset.

```
whole_body_bootstraps_clades <- boot.matrix(subsets_whole_body_clades,
bootstraps=number_bootstraps)
```

Compute disparity of each subset.

```
whole_body_disparity_clades <-
dispRity(whole_body_bootstraps_clades,metric=c(sum,ranges))
```

Test for statistical differences between nothosauroids and pachypleurosauroids.

```
test.dispRity(whole_body_disparity_clades,test=wilcox.test)
```

### 9.1.1. Plotting the results of the disparity per-clade analyses

Create a dataframe by extracting the bootstrapped data generated with the dispRity function (this will be used for the geom_jitter function in ggplot).

```
whole_body_disp_mat <-
data.frame(disp=double(),whole_body_disp_clades=character(),stringsAsFactors=
FALSE)
for (i in 1:length(whole_body_disp_clades)){
  whole_body_disp_mat[((i*number_bootstraps)-
number_bootstraps+1):(i*number_bootstraps),"disp"] <-
whole_body_disparity_clades$disparity[[i]][[2]][1,]
  whole_body_disp_mat[((i*number_bootstraps)-
number_bootstraps+1):(i*number_bootstraps), "Clades"] <-
names(whole_body_disp_clades)[i]
}

whole_body_disp_mat <- whole_body_disp_mat[,-2]
whole_body_disp_mat$Clades <- factor(whole_body_disp_mat$Clades, levels =
c("Pachypleurosauroidea", "Nothosauroidea"))
```

Visualize the result using ggplot.

```
whole_body_disparity_clades_fig <-
ggplot(data=whole_body_disp_mat,aes(Clades, disp))+
  geom_jitter(width=0.2,aes(color=Clades,alpha=0.5))+
  scale_color_manual(values=c('#6697cc','#F48656'))+
  geom_boxplot(aes(alpha=0.5),outlier.color = NA)+
  labs(y="Sum of ranges",x="")+
  labs(y="Sum of ranges",x="", title = "Whole body disparity per clade \n1000
boostraps; Wilcoxon test p value= 2.105e-10")+
  theme(plot.title=element_text(size=10), axis.text.x= element_text(size=
10,colour=c('#6697cc','#F48656')), legend.position="none",panel.grid.major =
element_blank(), panel.background = element_rect(fill="#d8d0f032"),
panel.grid.minor = element_blank())
```

## 9.2. Distribution of disparity per location

For these analyses, the number of bootstrap replications is set to 1000 as for the per-clade analyses.

Create a dataframe containing the scores on all axes of the PCoA and the location of each species. As only Augustasaurus has been found out from the Tethys ocean, it needs to be removed.

```
pcoa_whole_body_vectors_location <- as.data.frame(pcoa_whole_body$vectors)
pcoa_whole_body_vectors_location$location <- location
pcoa_whole_body_vectors_location$location <-
as.character(pcoa_whole_body_vectors_location$location)
pcoa_whole_body_vectors_location <- pcoa_whole_body_vectors_location[-34,] #
remove Augustasaurus
```

Create subsets (split) of the dataset according the clade of the species.

```
pcoa_whole_body_vectors_location <-
lapply(split(pcoa_whole_body_vectors_location,pcoa_whole_body_vectors_locatio
n$Location), rownames)
whole_body_subsets_location <-
custom.subsets(data=data.matrix(pcoa_whole_body$vectors),group=pcoa_whole_bod
y_vectors_location)
```

Bootstrap each subset.

```
whole_body_bootstraps_location <- boot.matrix(whole_body_subsets_location,
bootstraps=number_bootstraps)
```

Compute disparity of each subset.

```
whole_body_disparity_location <-
dispRity(whole_body_bootstraps_location,metric=c(sum,ranges))
```

Test for statistical differences between Eastern and Western Tethys by using a wilcoxon test.

```
test.dispRity(whole_body_disparity_location,test=wilcox.test)
```

### 9.2.1. Plotting the results of the regional disparity analyses

Create a data frame extracting the bootstraped data from dispRity (this will be used for the geom_jitter function in ggplot).

```
whole_body_disp_mat_location <-
data.frame(disp=double(),whole_body_disp_location=character(),stringsAsFactor
s=FALSE)
for (i in 1:length(whole_body_disp_location_pachy)){
  whole_body_disp_mat_location[((i*number_bootstraps)-
number_bootstraps+1):(i*number_bootstraps),"disp"] <-
whole_body_disparity_location$disparity[[i]][[2]][1,]
  whole_body_disp_mat_location[((i*number_bootstraps)-
number_bootstraps+1):(i*number_bootstraps), "locations"] <-
names(whole_body_disp_location)[i]
}

whole_body_disp_mat_location <- whole_body_disp_mat_location[,-2]

whole_body_disp_mat_location$location <-
factor(whole_body_disp_mat_location$locations, levels = c("Western Tethys",
"Eastern Tethys"))
```

Visualize the result using ggplot.

```
whole_body_disparity_location_fig <-
ggplot(data=whole_body_disp_mat_location,aes(locations, disp))+
  geom_jitter(width=0.2,aes(color=locations,alpha=0.5))+
  scale_color_manual(values=c('#ff6b77','#00c6cc'))+
  scale_x_discrete(breaks=c("Eastern_Tethys", "Western_Tethys"),
labels=c("Eastern Tethys", "Western Tethys"))+
  geom_boxplot(aes(alpha=0.5),outlier.color = NA)+
  labs(y="Sum of variances",x="", title = "Whole body regional disparity
\n1000 boostraps; Wilcoxon test p value= 2.795e-14")+
  theme(legend.position="none",  axis.text.x= element_text(size=
10,colour=c('#ff6b77','#00c6cc')), panel.grid.major = element_blank(),
panel.background = element_rect(fill="#d8d0f032"), panel.grid.minor =
element_blank())
```

## 9.3. Distribution of whole body nothosauroidean disparity per location

In this section, the disparity of Pachypleurosauroidea and Nothosauroidea will be respectively plotted per regional location.The number of bootstrap replications is set to 1000 as for the two previous analyses. The procedure shown here only concerns the distribution of pachypleurosauroids disparity in both eastern and Western Tethys and is the same for the nothosauroids according the correct selection of taxa.

Create a dataframe containing the scores on all axes of the PCoA and the location of each pachypleurosauroids.

```
pcoa_whole_body_vectors_location_pachy <-
pcoa_whole_body_vectors_location[1:17,] #selection of pachypleurosauroids
```

Create subsets (split) of the dataset according the location of species.

```
whole_body_disp_location_pachy <-
lapply(split(pcoa_whole_body_vectors_location_pachy,
pcoa_whole_body_vectors_location_pachy$location), rownames)

whole_body_subsets_location_pachy <-
custom.subsets(data=data.matrix(pcoa_whole_body$vectors[1:17,]),group=whole_b
ody_disp_location_pachy)
```

Bootstrap each subset.

```
whole_body_bootstraps_location_pachy <-
boot.matrix(whole_body_subsets_location_pachy, bootstraps=number_bootstraps)
```

Compute disparity of each subset.

```
whole_body_disparity_location_pachy <-
dispRity(whole_body_bootstraps_location_pachy,metric=c(sum,ranges))
```

Test for statistical differences between Eastern and Western Tethys by using a wilcoxon test.

```
test.dispRity(whole_body_disparity_location_pachy,test=wilcox.test)
```

### 9.3.1. Plotting the results of the pachypleurosauroidean regional disparity analysis

Create a data frame extracting the bootstraped data from dispRity (this will be used for the geom_jitter function in ggplot).

```
whole_body_disp_mat_location_pachy <-
data.frame(disp=double(),whole_body_disp_location_pachy=character(),stringsAs
Factors=FALSE)
for (i in 1:length(whole_body_disp_location_pachy)){
  whole_body_disp_mat_location_pachy[((i*number_bootstraps)-
number_bootstraps+1):(i*number_bootstraps),"disp"] <-
whole_body_disparity_location_pachy$disparity[[i]][[2]][1,]
  whole_body_disp_mat_location_pachy[((i*number_bootstraps)-
number_bootstraps+1):(i*number_bootstraps), "locations"] <-
names(whole_body_disp_location_pachy)[i]
}

whole_body_disp_mat_location_pachy <- whole_body_disp_mat_location_pachy[,-2]

whole_body_disp_mat_location_pachy$location <-
factor(whole_body_disp_mat_location_pachy$locations, levels = c("Western
Tethys", "Eastern Tethys"))
```

Visualize the result using ggplot.

```
whole_body_disparity_location_pachy_fig <-
ggplot(data=whole_body_disp_mat_location_pachy,aes(locations, disp))+
  geom_jitter(width=0.2,aes(color=locations,alpha=0.5))+
  scale_color_manual(values=c('#ff6b77','#00c6cc'))+
  geom_boxplot(aes(alpha=0.5),outlier.color = NA)+
  scale_x_discrete(breaks=c("Eastern_Tethys", "Western_Tethys"),
labels=c("Eastern Tethys", "Western Tethys"))+
  labs(y="Sum of ranges",x="", title = "Regional pachypleurosauroidean
overall disparity \n1000 boostraps; Wilcoxon test p value= 0")+
  theme(plot.title=element_text(size=13, colour ="#6697cc"), axis.text.x=
element_text(size= 13,colour=c('#ff6b77','#00c6cc')),
legend.position="none",panel.grid.major = element_blank(), panel.grid.minor =
element_blank(), panel.background = element_rect(fill="#d8d0f032"))
```

# 10. Convergence metrics

## 10.1 Using the metrics of Stayton

The Stayton convergence analyses will be computed by using the first-two and all axes of the PCoA generated with the whole body morphological dataset and also by using our timescaled phylogenetic tree (**whole_body_tree_mbl**) that we have generated above. The illustrated example here concerns the interclade convergence analysis between brevicaudosaurus jiyangshanensis and Wangosaurus brevirostris. If an analysis between other taxa is desired, we only need to mention their names

Choose the number of simulations.

```
nsim <- 1000
```

selection of the names of the taxa to be tested.

```
convtips_WB_BJ <-
c("Wangosaurus_brevirostris","Brevicaudosaurus_jiyangshanensis")
```

Values of the Ct1,Ct2,Ct3 and Ct4 metrics derived from the Stayton's metrics

```
Stayton_metrics_WB_BJ_2 <-
calcConvCt(phy=whole_body_tree_wango_notho_mbl,traits =
pcoa_whole_body$vectors[,1:2],focaltaxa=convtips_WB_BJ) # change
pcoa_whole_body$vectors [,1:2] pcoa_whole_body$vectors if you want to use all
axes of the PCoA

Stayton_metrics_WB_BJ_2_pvalue <- convSigCt(phy=whole_body_tree_mbl,traits =
pcoa_whole_body$vectors[,1:2],convtips_WB_BJ, nsim=nsim) # computation of
each pvalue under 1000 simulations
```

## 10.2 Convergence by using the method of Castiglione et al., 2019

These convergence analyses with the method developped by Castiglione et al., 2019 will be computed by using the first-two and all axes of the PCoA generated with the whole body

morphological dataset and also by using our timescaled phylogenetic tree (**whole_body_tree_mbl**) that we have generated above. The illustrated example here concerns the interclade convergence analysis between *brevicaudosaurus jiyangshanensis* and *Wangosaurus brevirostris*. If an analysis between other taxa is desired, we only need to mention their names by changing the object **convtips_**

Choose the number of simulations.

```
nsim <- 1000
```

Selection of the names of the taxa to be tested.

```
convtips_WB_BJ <-
c("Wangosaurus_brevirostris","Brevicaudosaurus_jiyangshanensis")
```

Create a vector that has the length of the number of tips in the phylogenetic tree and attribute the sate 'nostate' to all species.

```
states <- rep("nostate", length(whole_body_tree_mbl$tip.label));
names(states) <- whole_body_tree_mbl$tip.label
```

Only modify the state of focal species (you supposed to be convergent).

```
states[convtips_WB_BJ] <- "nothosauroidean_phenotype"
```

Run the convergence analysis between the two taxa.

```
conv_cast_WB_BJ <- search.conv(tree=whole_body_tree_mbl,
y=pcoa_whole_body$vectors[,1:2], state=states, declust=FALSE) # change
pcoa_whole_body$vectors.cor[,1:2] by pcoa_whole_body$vectors if you want to
use all axes of the PCoA
```

# 11. Distributions

## 11.1 Skull size distribution

### 11.1.1 Generate the log skull size distribution

```
skull_size_distribution <-
data.frame("skull_size"=c(log(split(Skull_size_to_plot$Skul.size,
Skull_size_to_plot$Clade)[[1]]),log(split(Skull_size_to_plot$Skul.size,
Skull_size_to_plot$Clade)[[2]])),"Clades_skull_size_to_plot"=c(rep("Pachypleu
rosauroidea",length(split(Skull_size_to_plot$Skul.size,Skull_size_to_plot$Cla
de)[[1]])),rep("Nothosauroidea",length(split(Skull_size_to_plot$Skul.size,Sku
ll_size_to_plot$Clade)[[1]]))))
```

### 11.1.2 Plotting the log skull size distribution

```
Skull_size_distribution_fig <- ggplot(skull_size_distribution,
aes(x=skull_size, fill= Clades_skull_size_to_plot,
color=Clades_skull_size_to_plot)) +
  geom_density(alpha=0.3,  lwd = 3, ) +
```

```
  annotate("text",x=3.75,y=0.75,label="Pachypleurosauroidea", lwd = 8,
color=clade_colours[2])+
  annotate("text",x=5.5,y=0.40,label="Nothosauroidea",  lwd = 8,
color=clade_colours[1])+
  labs(x="Ln skull size", y="density", title= "Distribution of skull size")+
  scale_fill_manual(values=c('#6697cc','#F48656'))+
  scale_color_manual(values= c('#6697cc','#F48656'))+
  theme(legend.position = "none", panel.background =
element_rect(fill="#EDEDED"), panel.grid.major = element_blank(),
panel.grid.minor = element_blank())
```

#### 11.1.3 T-test between pachypleurosauroids and nothosauroids

```
t.test(split(Skull_size_to_plot$Skul.size,Skull_size_to_plot$Clade)[[1]],
split(Skull_size_to_plot$Skul.size,morpho_full$Clade)[[2]])
```

### 11.2 Posterior skull width distribution

#### 11.1.1 Generate the log posterior skull width distribution

```
Posterior_skull_width_clade <- data.frame("width" =
c(log(split(Posterior_skull_width$Posterior_skull_width,
Posterior_skull_width$Clade)[[1]]),
log(split(Posterior_skull_width$Posterior_skull_width,
Posterior_skull_width$Clade)[[2]])),"clade_width"=c(rep("Pachypleurosauroidea
",length(split(Posterior_skull_width$Posterior_skull_width,
Posterior_skull_width$Clade)[[1]])),rep("Nothosauroidea",length(split(Posteri
or_skull_width$Posterior_skull_width, Posterior_skull_width$Clade)[[2]]))))
```

#### 11.1.2 Plotting the log skull size distribution

```
Skull_width_fig <- ggplot(Posterior_skull_width_clade, aes(x=width, fill=
clade_width, color=clade_width)) +
  geom_density(alpha=0.3,  lwd = 3, ) +
  annotate("text",x=3.4,y=0.67,label="Pachypleurosauroidea", lwd = 8,
color=clade_colours[2])+
  annotate("text",x=4.7,y=0.43,label="Nothosauroidea",  lwd = 8,
color=clade_colours[1])+
  labs(x="Ln skull width", y="density", title= "Distribution of skull
width")+
  scale_fill_manual(values= c('#6697cc','#F48656'))+
  scale_color_manual(values= c('#6697cc','#F48656'))+
  theme(legend.position = "none", panel.background =
element_rect(fill="#EDEDED"), panel.grid.major = element_blank(),
panel.grid.minor = element_blank())
```

#### 11.1.3 T-test between pachypleurosauroids and nothosauroids

```
t.test(split(Posterior_skull_width$Posterior_skull_width,
Posterior_skull_width$Clade)[[1]],
split(Posterior_skull_width$Posterior_skull_width,Posterior_skull_width$Clade
)[[2]])
```
